# Progress and Pitfalls in the Quest for Effective SARS-CoV-2 (COVID-19) Vaccines

**DOI:** 10.3389/fimmu.2020.579250

**Published:** 2020-10-02

**Authors:** Katie L. Flanagan, Emma Best, Nigel W. Crawford, Michelle Giles, Archana Koirala, Kristine Macartney, Fiona Russell, Benjamin W. Teh, Sophie CH Wen

**Affiliations:** ^1^Department of Infectious Diseases, Launceston General Hospital, Launceston, TAS, Australia; ^2^Faculty of Health Sciences and School of Medicine, University of Tasmania, Launceston, TAS, Australia; ^3^School of Health and Biomedical Science, Royal Melbourne Institute of Technology (RMIT) University, Melbourne, VIC, Australia; ^4^Department of Immunology and Pathology, Monash University, Melbourne, VIC, Australia; ^5^Department of Paediatric Infectious Diseases, Starship Children's Hospital, Auckland, New Zealand; ^6^Department of Paediatrics: Child and Youth Health, University of Auckland, Auckland, New Zealand; ^7^Department of Paediatrics, University of Melbourne, Melbourne, VIC, Australia; ^8^Murdoch Children's Research Institute, Royal Children's Hospital Immunisation Service, Melbourne, VIC, Australia; ^9^Department of Obstetrics and Gynaecology, Monash University, Melbourne, VIC, Australia; ^10^Infectious Diseases Unit, Alfred Health, Melbourne, VIC, Australia; ^11^Department of Child and Adolescent Health, University of Sydney, Sydney, NSW, Australia; ^12^National Centre for Immunisation Research & Surveillance (NCIRS), Sydney, NSW, Australia; ^13^Department of Infectious Diseases, Nepean Hospital, Sydney, NSW, Australia; ^14^Department of Infectious Diseases, Peter MacCallum Cancer Centre, Melbourne, VIC, Australia; ^15^Sir Peter MacCallum Department of Oncology, University of Melbourne, Melbourne, VIC, Australia; ^16^Infection Management Prevention Services, Queensland Children's Hospital, South Brisbane, QLD, Australia; ^17^University of Queensland Centre for Clinical Research (UQCCR), University of Queensland, Brisbane, QLD, Australia

**Keywords:** antibody dependent enhancement (ADE), adverse events of special interest (AESI), bacillus Calmette-Guérin (BCG), cell mediated immunity, Coalition for Epidemic Preparedness Innovations (CEPI), innate immunity, neutralizing antibodies, spike protein

## Abstract

There are currently around 200 SARS-CoV-2 candidate vaccines in preclinical and clinical trials throughout the world. The various candidates employ a range of vaccine strategies including some novel approaches. Currently, the goal is to prove that they are safe and immunogenic in humans (phase 1/2 studies) with several now advancing into phase 2 and 3 trials to demonstrate efficacy and gather comprehensive data on safety. It is highly likely that many vaccines will be shown to stimulate antibody and T cell responses in healthy individuals and have an acceptable safety profile, but the key will be to confirm that they protect against COVID-19. There is much hope that SARS-CoV-2 vaccines will be rolled out to the entire world to contain the pandemic and avert its most damaging impacts. However, in all likelihood this will initially require a targeted approach toward key vulnerable groups. Collaborative efforts are underway to ensure manufacturing can occur at the unprecedented scale and speed required to immunize billions of people. Ensuring deployment also occurs equitably across the globe will be critical. Careful evaluation and ongoing surveillance for safety will be required to address theoretical concerns regarding immune enhancement seen in previous contexts. Herein, we review the current knowledge about the immune response to this novel virus as it pertains to the design of effective and safe SARS-CoV-2 vaccines and the range of novel and established approaches to vaccine development being taken. We provide details of some of the frontrunner vaccines and discuss potential issues including adverse effects, scale-up and delivery.

## Introduction

The recent emergence of severe acute respiratory syndrome coronavirus-2 (SARS-CoV-2), the cause of coronavirus disease (COVID-19), is wreaking havoc due to widespread dissemination throughout the world. On 11 March 2020, WHO formally declared that a global pandemic and by the end of August 2020, almost 25 million cases and over 800,000 deaths had been reported worldwide involving all continents, except Antarctica (1). Strategies to identify cases and limit spread by widespread testing and physical distancing have been challenging to implement, healthcare and public health systems have been overwhelmed, resulting in continued escalation in many countries and profound effects on lives and livelihoods. While the majority of people are either asymptomatic or experience a mild respiratory infection, ~20% of cases are more severe and require hospital admission (2). Reported mortality rates vary by geographic region, ranging from almost 20% in France to <1% in many other countries (3). This wide discrepancy suggests selection bias due to differences in local testing strategies and capacity, consistent with differences in health system capacity, population demographics and other health determinants. Certain groups such as the elderly and those with particular comorbidities are more likely to die of COVID-19 (3). Healthcare workers in particular have experienced significant morbidity and mortality from COVID-19 (4), causing clear psychological impacts and threatening delivery of healthcare services (5). There is no known effective treatment for this virus and currently no available vaccine, with the result being that SARS-CoV-2 continues to spread throughout the virus naïve population of the world. The urgent need for effective SARS-CoV-2 vaccines cannot be overstated. Immunization can not only protect individuals but also, if provided to enough people in a timely way with (even) partially protective vaccines, induce sufficient herd immunity to curtail the spread of this virus and reduce morbidity and mortality across the globe.

The race to develop safe and effective vaccines has seen SARS-CoV-2 candidate vaccines developed at a scale and pace never imagined before: currently almost 200 potential vaccines are in various stages of development (6–8). A range of vaccine design approaches and platforms have been employed. However, since >95% of candidate vaccines typically fail it is expected that the eventual number of successful vaccines may be only be a handful. They may also become available in different time frames and suitable for use in different populations. Most vaccine candidates are currently in preclinical trials, but a number have entered phase 1 or phase 1/2 studies, with plans to rapidly scale up to phase 2 and 3. Trials are being conducted at “pandemic speed” (8) and using novel designs. This early success has already seen cooperation and collaboration as well as significant funding across the globe. For example, the Coalition for Epidemic Preparedness Innovations (CEPI), a not-for-profit global coalition launched in 2017 to deal with the worldwide threat of epidemic outbreaks, is playing a pivotal role in supporting many of the frontrunner vaccines (9).

Herein, we review what is currently known about the immune response to SARS-CoV-2 and the various vaccine platforms being used to develop the SARS-CoV-2 vaccines. Understanding the mechanism of action of the various candidate vaccines is the key focus of this review. We also discuss potential challenges at the immunological level, assessment of vaccine safety and scale-up and delivery.

## SARS-CoV-2 Structure and Function

Coronaviruses are enveloped positive-sense single stranded RNA viruses belonging to the *Coronaviridae* family. They infect birds and mammals causing a range of symptoms from respiratory to gastrointestinal disease (10). A number of relatively common seasonal coronaviruses are known to infect humans (11), causing mild respiratory illness (“the common cold”). Two previous lethal human coronavirus diseases, namely severe acute respiratory syndrome (SARS caused by SARS-CoV-1) (12) and Middle East respiratory syndrome (MERS caused by MERS-CoV) (7) arose in 2002 and 2012, respectively. They have a high mortality rate of ~9 and 40%, respectively, but fortunately, neither reached pandemic levels. SARS-CoV-1 was able to be contained by public health measures to prevent human-to human transmission and then disappeared before vaccine development had progressed significantly.

**Figure 1:**
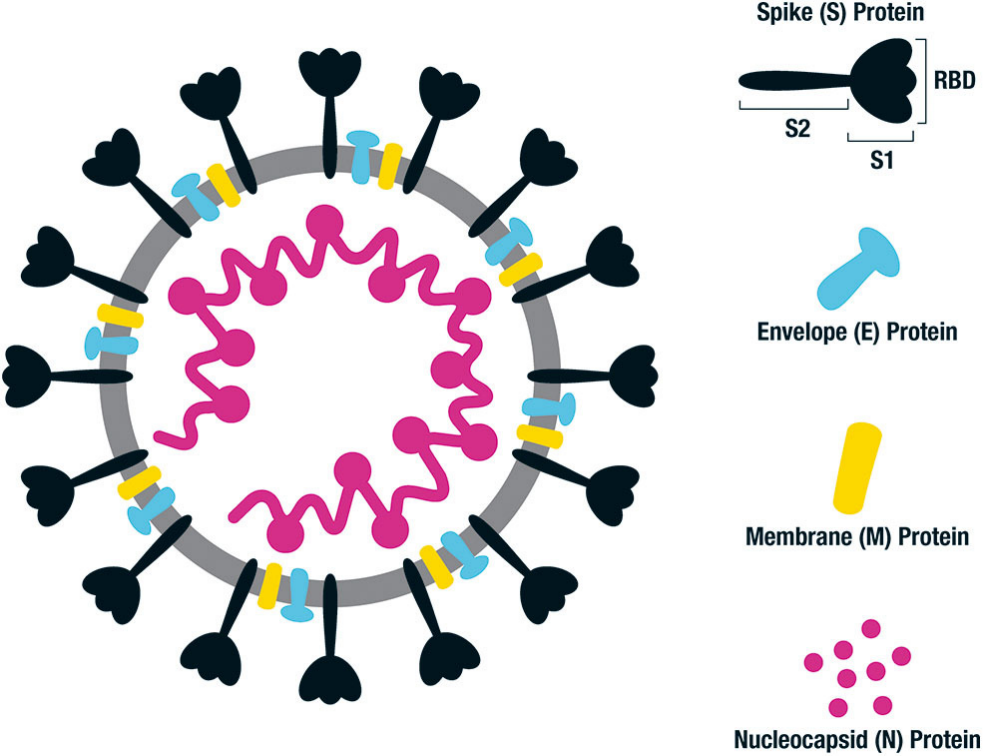
Structure of SARS-CoV-2 and key antigenic components. Illustration of SARS-CoV-2 which is a single stranded RNA virus. The key antigenic components being targeted in vaccine design are shown on the right, consisting of the spike (S), envelope (E), membrane (M), and nucleocapsid (N) proteins. The main emphasis for human vaccines is based on the spike (S) protein, consisting of an S1 binding region and S2 fusion and cell entry region. The S1 domain contains the receptor binding domain (RBD) responsible for binding to the ACE2 receptor on the surface of host cells. Following fusion, the S protein sheds the S1 region and undergoes a dramatic structural change to its post-fusional state in order for the virus to enter the host cells.

The genome of SARS-CoV-2 encodes for the structural proteins spike (S), envelope (E), membrane (M) and nucleocapsid (N) as well as a number of accessory and non-structural proteins (Figure 1) (13). The M and N proteins give the virion its shape, and the S proteins appear as spikes on the viral surface giving it a solar corona appearance. The S glycoprotein (spike) exists in trimeric form and is the structure by which binding to the host cells occurs. The virus receptor, angiotensin-converting enzyme 2 (ACE2), expressed on the surface of multiple human cells is engaged via the receptor binding domain (RBD), which is part of the S1 subunit of the S glycoprotein (14) (Figure 1). The S2 subunit consists of two heptad repeat fusion regions, HR1 and HR2, responsible for membrane fusion and cell entry. The RBD domain can either be down and buried or rotated up and primed for ACE2 binding (15). The hidden down state is the predominant state of the S protein trimer in SARS-CoV-2, and likely a mechanism the virus uses to hide the entry epitopes and evade the host immune response (16). Two S trimers can concurrently bind to an ACE2 dimer. Following S protein binding in its prefusion state, the S1 subunit is cleaved and shed permitting the S2 dramatic conformational changes which constitute the post-fusion state required for viral entry (17, 18).

## Immune Response to SARS-CoV-2

### Innate Immunity to COVID-19: Protection or Hyper-Activation?

**Figure 2:**
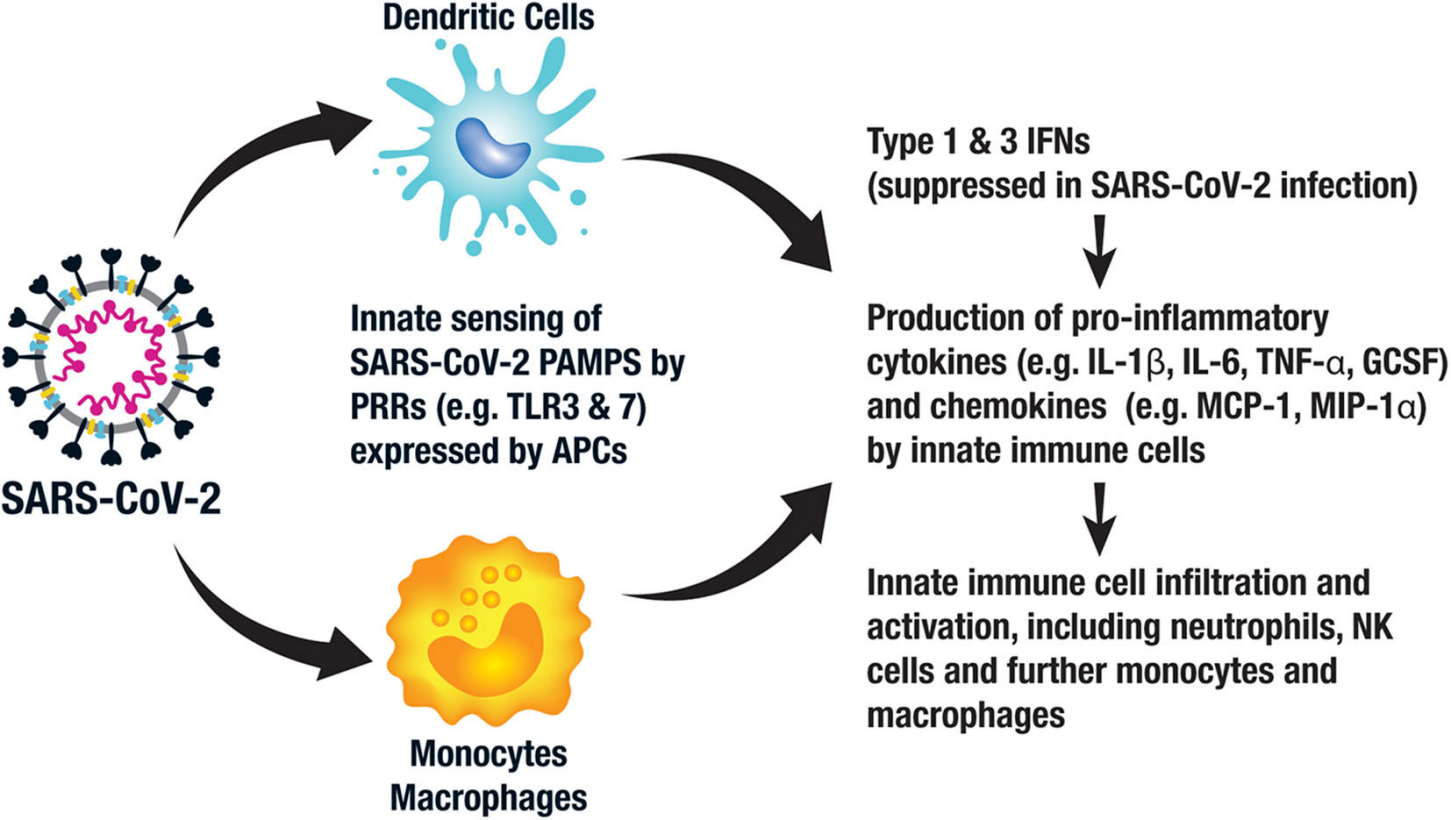
Key components of the innate immune response to SARS-CoV-2. Antigen presenting cells (APCs), such as monocytes, macrophages, and dendritic cells (DCs), recognize pattern associated molecular patterns (PAMPs) expressed by SARS-CoV-2 via their pattern recognition receptors (PRRs), such as toll-like receptor (TLR) 3 and 7. This activates intracellular signaling pathways leading to the expression of type 1 and 3 interferons (IFNs), which in turn activate innate immune cells to produce pro-inflammatory cytokines and chemokines. This leads to an influx and activation of neutrophils, further APCs and other innate immune cells, such as natural killer (NK) cells.

SARS-CoV-2 stimulates an innate immune response via pattern associated molecular patterns (PAMPs) expressed by the virus, which in the case of SARS-CoV-2 consists of viral RNA and its intermediates produced during replication (19). These conserved PAMPs stimulate multiple immune response pathways via pattern recognition receptors (PRRs), including sensing by endosomal Toll-like receptors (TLRs) 3 and 7, cytosolic retinoic acid-inducible gene 1 (RIG-1) and melanoma differentiation-associated protein 5 (MDA5). Local tissue damage in the lungs also releases damage-associated molecular patterns (DAMPS) further contributing to local inflammation. The resultant inflammatory response provides immediate antiviral immunity via activation of antiviral type 1 and 3 interferon (IFN) pathways leading to an upregulation of inflammatory cytokines such as interleukin 6 (IL-6) and IL-1β, further recruiting neutrophils, other innate immune cells and stimulating anti-SARS-CoV-2 adaptive memory T cells and B cells (20) (Figure 2).

There is still much to understand about the immune response to SARS-CoV-2 and the immunological differences in those with mild as compared to severe infection. Emerging data suggest that the innate response to SARS-CoV-2 is aberrant (21, 22). For example, the early type I and III interferon responses are relatively suppressed by SARS-CoV-2, an immune evasion strategy employed by the virus, leading to early failure to control the virus. Furthermore, uncontrolled local inflammation, or what has been described as “cytokine storm,” leading to tissue damage with inflammatory cell infiltration and the acute respiratory distress syndrome (ARDS) is thought to characterize late stage, severe manifestations of COVID-19 (23–25). For example, patients with severe COVID-19 had higher levels of IL-6, IL-2R, IL-10, and tumor necrosis factor alpha (TNF-α) than those with moderate disease, the former of which correlated with clinical severity and death (26, 27). Those COVID-19 patients requiring ICU admission demonstrated greater plasma levels of IL-2, IL-7, IL-10, granulocyte colony-stimulating factor (GCSF), interferon-inducible protein 10 (IP-10), monocyte chemoattractant protein-1 (MCP-1), macrophage inflammatory protein-1α (MIP-1α) and TNF-α than the non-ICU COVID-19 patients, all supporting enhanced innate immunity in the sicker patients (28). Of note, the IL-6 levels described in COVID-19 patients are a log-fold lower than those described in classic cytokine storm (29). Furthermore, IL-10 is an immunosuppressive cytokine suggesting dysregulated immune homeostasis rather than pure inflammation. Upregulated chemoattractant chemokines further recruit inflammatory cells including neutrophils, macrophages, natural killer (NK) cells and T cells resulting in further immunopathology (21) (Figure 2).

### Induction of SARS-CoV-2 Specific Neutralizing Antibodies

**Figure 3:**
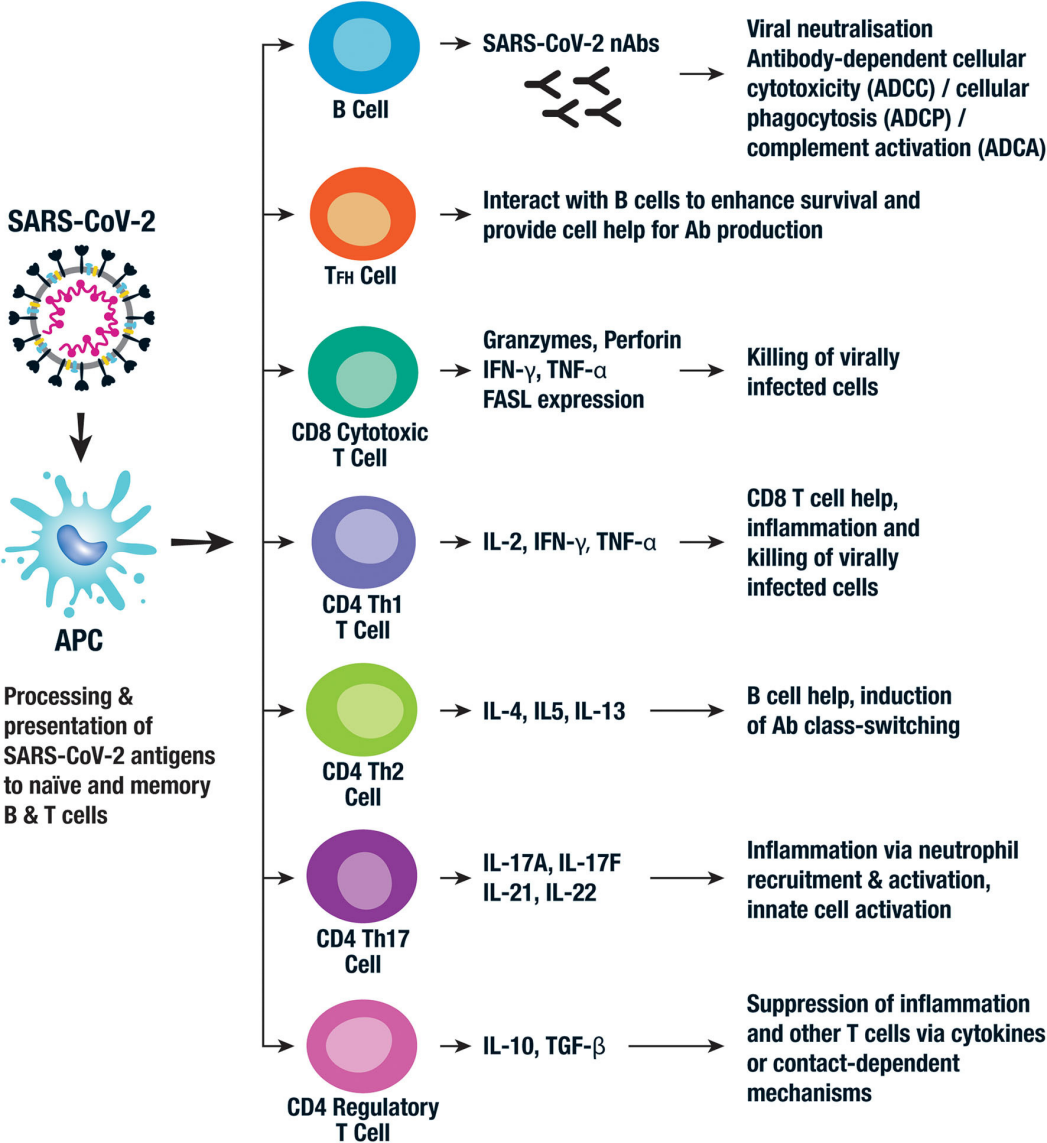
Key components of the adaptive immune response to SARS-CoV-2. The adaptive immune response is activated following viral uptake and antigen processing by a range of APCs. The APCs present viral antigen to B cells which then differentiate into antibody producing plasma cells. The neutralizing antibodies (nAbs) then bind to key viral proteins, such as the spike protein, and neutralize their activity. Other Ab-mediated antiviral functions include antibody dependent cellular cytotoxicity (ADCC), antibody dependent cellular phagocytosis (ADCP), and antibody dependent complement activation (ADCA). Cytotoxic CD8^+^ T cells kill virally infected cells via the production of granzymes and perforin and the expression of Fas ligand (FasL), all of which mediate cellular apoptosis. A series of CD4^+^ T cell populations are involved in the adaptive cellular response to SARS-CoV-2. Follicular helper T cells (T_FH_) and Th2 CD4^+^ T cells both provide help for B cell antibody production. Th1 and Th17 CD4^+^ T cells are also thought to play a role in the inflammatory response and viral killing. CD4^+^ regulatory T cells have been implicated with an immunoregulatory role in SARS-CoV-2 infection via the production of anti-inflammatory cytokines and contact-mediated cellular suppression. Whether CD8^+^ Tregs and Bregs play a role is not currently known.

Antibodies (Abs) produced by activated B cells play a key role in anti-viral immunity via several mechanisms including viral neutralization, antibody-dependent cellular cytotoxicity (ADCC), antibody-dependent cellular phagocytosis (ADCP), and antibody-dependent complement activation (ADCA) (Figure 3). It is thought that generation of high levels of neutralizing antibodies (nAbs) against SARS-CoV-2 are required for a successful human vaccine (18). However, some patients recover without producing high levels of nAbs and those with severe disease may experience an early rise in nAbs (30). Nevertheless, most vaccine efforts are focused on the induction of nAbs to the S protein in order to block attachment of RBD to the ACE2 receptor on host cells (31). As mentioned before, this domain is hidden in the S protein's prefusion state, presenting challenges to the success of RBD-based vaccines (16). For this reason, some groups have focused on eliciting nAbs to the less immunogenic S2 subunit of the spike protein (16) and SARS-CoV-2 vaccines based on other antigens, including the N protein, are also being developed.

A recent report from the US describes outcomes among 35,322 COVID-19 patients, many of whom had critical illness, transfused with the plasma from people who have recovered from COVID-19 (convalescent plasma [CP]) (32). Early CP infusion within 3 days of illness had a lower 7-day mortality than those treated after 4 or more days (8.7% vs. 11.9%). In this uncontrolled study, those who received higher levels of IgG Abs in the transfused plasma had a better mortality outcome. These data, alongside results from another uncontrolled study showing recovery in severely unwell COVID-19 patients treated with CP, lends support to the notion that naturally acquired Abs can be protective (33). However, other plasma factors such as cytokines, defensins and other non-specific Abs may also play a protective role in these studies (34). SARS-CoV-2-specific nAbs recovered from infected humans also protected Syrian hamsters and rhesus macaques in challenge studies (35, 36). Ideally, a SARS-CoV-2 vaccine would induce long-lasting Abs, but it is not yet known how long specific Abs persist in SARS-CoV-2 infected individuals or how long they would persist after vaccination. Indeed, several recent studies indicate rapid waning of SARS-CoV-2 nAbs in some individuals following natural infection, although others maintained high levels to 60–94 days (37, 38), raising concerns about the persistence of nAbs post-immunization; whether nAbs plateau or continue to decline over time is yet to be determined. By contrast, nAbs to SARS and MERS have been detected up to 2–3 years after infection in human survivors (39) and a recent study reports nAbs 17 years after SARS infection, suggesting that long-lasting coronavirus-specific nAbs can be induced in some instances (40) so hopefully Ab mediated immunity to SARS-CoV-2 will be equally long-lasting. Importantly, class-switched IgG memory B cells to S and RBD have been demonstrated in COVID-19 patients confirming the generation of B cell memory which can provide a rapid recall response on subsequent SARS-CoV-2 exposure (41). Furthermore, we do not yet know what level of nAbs are required for protection (42). The standardization of a range of assays to support vaccine studies, such as viral neutralization assays, to enable comparison of different vaccine candidates in different populations will be key to facilitating vaccine development, an issue which represents a current focus of the WHO (43).

### T Cell Adaptive Immunity: Protection, Immune Suppression or Disease Enhancement?

Processing and presentation of SARS-CoV-2 epitopes by antigen presenting cells (APCs) via human leukocyte antigen (HLA) class I leads to activation of naïve CD8^+^ T cells which differentiate into cytolytic effectors (cytotoxic T lymphocytes [CTL]) that kill virus-infected cells. Activation of T helper 1 (Th1) CD4^+^ T cells via viral peptides presented on HLA class II alleles further enhances the CD8^+^ T cell response, while HLA class II-restricted follicular helper (T_FH_) and Th2 cells enhance virus-specific antibody production (Figure 3). Effective viral clearance therefore requires a combination of CD8^+^ CTL and CD4^+^ T cell mediated enhancement of B cell and CD8^+^ T cell responses (20). Pro-inflammatory Th17 also play a role as evidenced by their high frequency in lung biopsy specimens of COVID-19 patients (44) (Figure 3). Severe viral disease, associated with an over exuberant T cell response, leads to local inflammation and toxicity, thus activation of immune modulatory factors or checkpoints is crucial in limiting the damage. Indeed, regulatory T cells (Tregs) play a key role in the control of inflammation and immunopathology in other coronavirus infections (45), but this has yet to be investigated for SARS-CoV-2 (Figure 3). Most activated T cells subsequently apoptose and die, but some persist and provide long-term virus-specific T cell immunity. Natural infection with SARS-CoV-1 induced long-lasting memory T cells up to 6 years after infection (46) and memory T cells persisted to 2 years after MERS infection (47), supporting the potential for long-term persistence in SARS-CoV-2 infection. These should provide protective immunity on subsequent exposure to the pathogen, the ultimate goal of an effective vaccine.

Severe COVID-19 patients exhibit immunological features associated with T cell anergy or exhaustion. For example, the lungs of severe COVID-19 patients contain high levels of CD8^+^ T cells expressing classic markers and genes of exhaustion (29), while patients who have recovered from moderate or severe COVID-19 seem to have robust memory T cell formation (48). These infiltrating cells also express high levels of markers of activation and cytotoxicity. Other studies have similarly demonstrated that more activated and, in some cases, more exhausted T cells develop in symptomatic COVID-19 patients compared to levels during the prodromal stage (49). Those who experience mild-moderate disease maintain their lymphocyte counts and have more polyfunctional T cells; while studies in those with severe disease are contradicting, reporting both lower and higher CD8^+^ T cell cytotoxicity (49). Severe disease is associated with profound lymphopaenia lending support to the theory that the disease also causes immunosuppression, although lymphocyte trafficking to the site of infection may also be responsible (49). A biphasic T cell response has therefore been proposed, characterized by an early CD8^+^ cytotoxic T cell response to control the virus followed by T cell exhaustion and depletion as a result of viral persistence over many weeks in some patients. This model could account for poorer outcomes in the elderly who have diminished T cell repertoires and better outcomes in children who have a diverse and abundant naïve T cell pool (29). Nevertheless, the likely early protective role for CD4^+^ and CD8^+^ T cells in SARS-CoV-2 clearance suggests that their induction should be exploited in SARS-CoV-2 vaccine development strategies.

## Knowledge From SARS and MERS Vaccine Development

No vaccine is licensed or available for SARS or MERS, however, considerable preclinical development studies have been performed. A range of vaccine development approaches were explored, including live attenuated vaccines and inactivated vaccines (such as inactivated whole virus), soluble protein vaccines, viral vectors, nanoparticles, and DNA vaccines; most of these platforms are also being utilized for SARS-CoV-2, as discussed below (50, 51). Most of the SARS and MERS subunit candidate vaccines have targeted the S glycoprotein and been examined in studies conducted in animal models, although N, M, and E protein-based vaccines have also been tested (20). However, the fact that animal models, including mice and non-human primates, display only limited clinical manifestations of SARS and MERS, that it is not usually lethal, severely limit the translation of results into humans. To overcome this, animal adapted strains and transgenic animal models of severe disease have been developed (20, 52).

In virus challenge studies in animals, high titers of nAbs to both SARS-CoV-1 and MERS virus correlated with protection (51, 53). Relatively few studies have investigated the protective role of T cells, and while some correlate CD4^+^ and CD8^+^ T cell responses with protection, adoptive transfer and T cell depletion assays in mice suggest they are not necessary for protection in mice immunized with a DNA-based SARS vaccine (54). Furthermore, vaccination of mice with SARS and MERS candidate vaccines has been shown to result in Th2 lung pathology with eosinophilic infiltration following wild-type virus challenge (54–57). This immunopathological effect has been associated with whole virus vaccines with and without adjuvant and linked with responses to the N protein in particular; S protein-based vaccines may have a lesser effect. An N protein-based DNA SARS vaccine also elicited a delayed type hypersensitivity reaction in mice, further cautioning against using this protein for SARS-CoV-2 vaccines (57). Furthermore, S-based vaccines appear more immunogenic and protective in these studies and the duration of protection has been shown in challenge studies to last 3–12 months in mice immunized with a vesicular stomatitis virus (VSV)-based SARS vaccine and animals immunized with DNA and/or protein-based MERS vaccines (23, 58). Of note, in the former study, protection against viral challenge was complete in younger mice but limited in senescent mice, highlighting potential issues with successful vaccination in older individuals (59).

The only SARS vaccines to reach human phase 1 clinical trials were based on inactivated virus, soluble S glycoprotein and DNA approaches (52). MERS vaccines tested in human phase 1 clinical trials include a DNA-based vaccine alone (60); DNA in combination with adenovirus or modified vaccinia Ankara (MVA) viral vectors as a prime-boost strategy (52, 61); and a chimp adenovirus vaccine (62). Efficacy against disease has not been tested in humans since it has not been deemed ethical to perform challenge studies with lethal viruses for which there is no effective treatment.

## Viral Sequencing and Immunoinformatics to Inform Rational Vaccine Design

The first SARS-CoV-2 genome was isolated from patients with pneumonia from Wuhan, China in early 2020 and identified as a novel human coronavirus (63, 64). Since that time, multiple viruses have been isolated and sequenced from patients throughout the world providing an understanding of the evolutionary capacity and diversity of this pathogen (63). Structural genomics and molecular interaction analysis (interactomics) can be used to rapidly identify putative functional sites, facilitating rational vaccine design by identifying potential B and T cell epitope regions of key viral proteins (65). Several groups have used computational programs and immunoinformatics to predict CD4^+^ and CD8^+^ T cell and B cell epitopes across multiple SARS-CoV-2 antigens from pathogen sequence data to design novel SARS-CoV-2 vaccines (66, 67). The genetic diversity and stability of these regions over time can then be characterized, and cross-reactivity predicted and examined to ensure a vaccine provides cross-strain immunity. The S gene of human coronaviruses is well-known to undergo genetic drift which could compromise SARS-CoV-2 vaccines based on this region (68, 69). Reassuringly, comparisons between the sequences of known SARS-CoV-1 B and T cell epitopes from the N and S proteins have been shown that some of the epitopes map identically to proteins from SARS-CoV-2 (70). Importantly, these epitopes showed no mutations among 120 available SARS-CoV-2 sequences suggesting that they reside in stable parts of the S protein. The T cell epitopes also covered a broad range of HLA alleles and could therefore be future targets for vaccine design.

One issue with this approach is that the epitopes will be specific for human HLA alleles, making it difficult to test them for protective efficacy in animal models. One way to overcome this problem is to use humanized mouse models which have functional human immune systems (71), bearing in mind that mice can also be modified to express the ACE2 receptor (72). Also, there are often animal homologs of human CD4 and CD8 T cell epitopes so it may still be possible to screen for efficacy. However, it should be borne in mind that due to the urgent need to develop an effective SARS-CoV-2 vaccine, animal challenge data are not a mandatory pre-requisite to progression to clinical trials; nevertheless, ideally these should be conducted as part of the vaccine development strategy. The recent development of a laboratory-adapted SARS-CoV-2 strain that is pathogenic in mice, provides an ideal animal challenge model for testing the efficacy of SARS-CoV-2 candidate vaccines in mice (73).

## Different Vaccine Approaches Used in COVID-19 Vaccine Development

**Figure 4:**
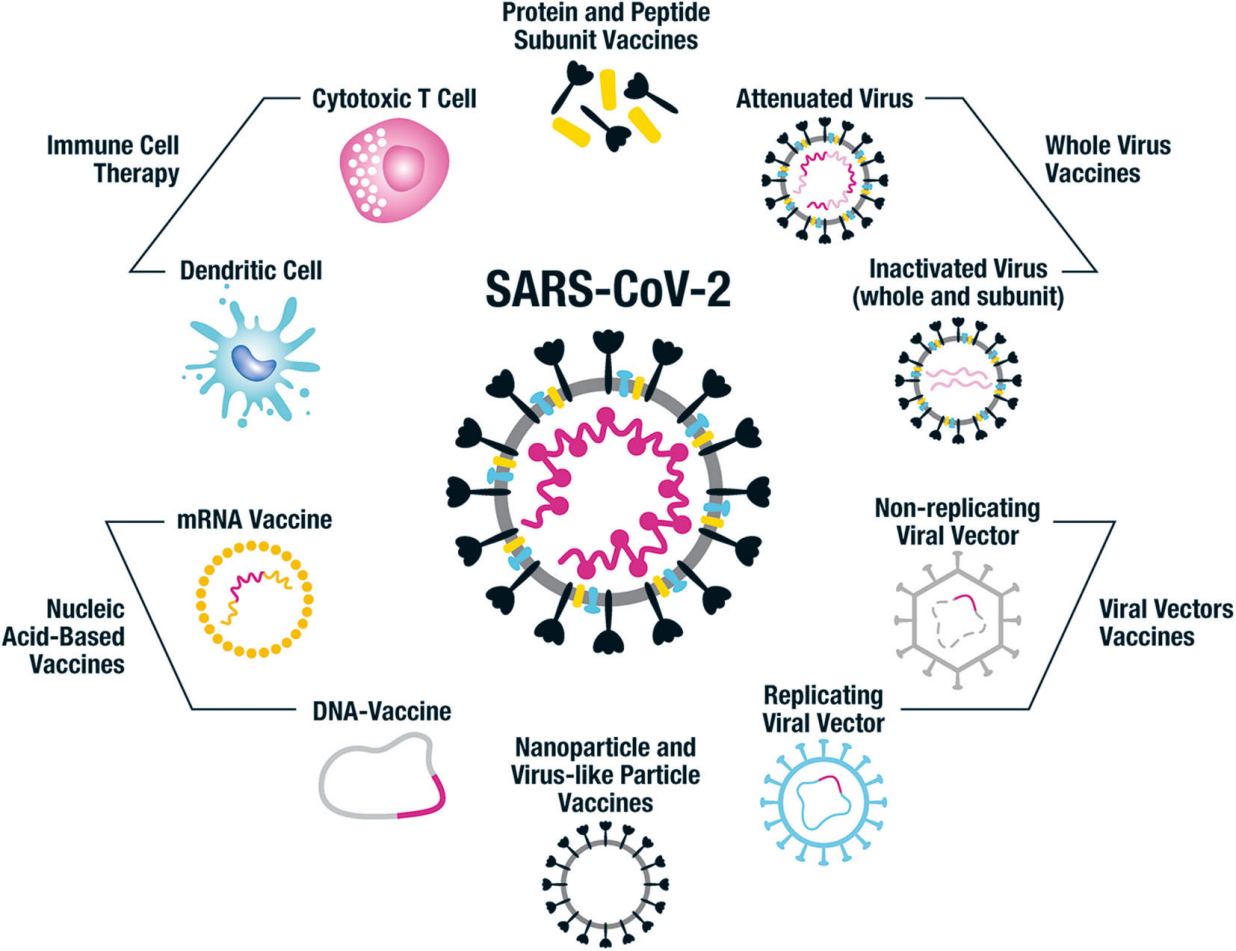
Vaccine platforms being employed for SARS-CoV-2 vaccine design. This figure illustrates the different vaccine approaches being taken for the design of human SARS-CoV-2 vaccines. Whole virus vaccines include both attenuated and inactivated forms of the virus and subunits of inactivated virus can also be used. Protein and peptide subunit vaccines are usually combined with an adjuvant in order to enhance immunogenicity. The main emphasis in SARS-CoV-2 vaccine development has been on using the whole spike protein in its trimeric form or components of it, such as the RBD region. Multiple non-replicating viral vector vaccines have been developed, particularly focused on adenovirus; while there has been less emphasis on the replicating viral vector constructs. Nucleic acid-based approaches include DNA and mRNA vaccines, often packaged into nanocarriers such as virus-like particles (VLPs) and lipid nanoparticles (LNPs). Nanoparticle and VLP vaccines can also have antigen attached to their surface or combined in their core. The immune cell therapy approach uses genetically modified SARS-CoV-2-specific cytotoxic T cells and dendritic cells expressing viral antigens to protect against SARS-CoV-2 infection. Each of these vaccine approaches has benefits and disadvantages in terms of cost and ease of production, safety profile and immunogenicity, and it remains to be seen which of the many candidates in development protect against COVID-19.

Vaccine technology has been advancing rapidly and there is a plethora of approaches to constructing SARS-CoV-2 vaccines. These range from the original technique employed centuries ago in the smallpox vaccine development of using modified or killed whole organism, to protein and peptide-based vaccines, nucleic acid DNA and RNA vaccines and nanoparticle constructs. Each approach has unique advantages and disadvantages including varying time to development and scalability, cost, stability, as well as anticipated safety and immunogenicity profiles; all approaches are represented among preclinical and clinical trials (58–60) (Figure 4).

### The Need for Vaccine Adjuvants

Some vaccine candidates, particularly protein-based vaccines, require an adjuvant to boost their immunogenicity (74). To date, very few adjuvants have been licensed for human use since the original adoption of aluminum salts (alum) from the early 1920s, which bias immunity toward a Th2 response (75). Several of the newer adjuvants consist of Toll-like receptor agonists to stimulate innate immunity combined with alum, such as AS04 which is based on the TLR4 ligand monophosphoryl lipid A (MPL). Oil-in-water emulsion adjuvants including MF59 and AS03 are stronger adjuvants than alum and have been adopted in several licensed influenza vaccines. The adjuvant AS01, present in the malaria vaccine RTS,S, combines MPL and a saponin QS-21 (75). A wide variety of adjuvant approaches are being taken with the various SARS-CoV-2 vaccines in development, including those mentioned above, some of which are described in the relevant sections below (76).

### Different Routes of Vaccination

Many vaccines are given via the intramuscular route, including the majority of the current COVID-19 vaccines in development (Table 1). However, since SARS-CoV-2 causes infection via the respiratory tract, a vaccine targeting the mucosal immune system might be preferable (77). Mucosal vaccines have the advantage of being needle-free and thus practical for mass-administration, but immune tolerance induction can be an issue. The formulation, including the use of mucosal adjuvants, is therefore important to ensure stimulation of adequate local and systemic immunity (78).

**Table 1:** Vaccines approaches being taken and the number of candidate vaccines in clinical and pre-clinical trials (20 August 2020).

**Vaccine platform**	**Construct details**	**Number in pre-clinical trials**	**Number in clinical trials**
Live attenuated virus	Codon-pair deoptimized live attenuated vaccines	3	0
Inactivated whole virus	Some combined with alum or CpG 1018 adjuvant	9	5
Protein/peptide subunit	Recombinant whole S protein, RBD or S1; 1 molecular clamp stabilized; 1 based on N protein; nanoparticle/VLP; peptides in LNP; 1 li-key peptide-based; OMV-based With or without adjuvant and/or fused with Fc subunit of IgG Most intramuscular delivery; 1 microneedle array, 1 oral construct	50	8
Non-replicating viral vectors	Chimp adenovirus 1; human adenovirus (Ad5, Ad 26); adeno-associated virus (AAV); influenza virus (H1N1); modified vaccinia Ankara (MVA); parainfluenza virus 5 (PIV5); rabies virus; Sendai virus With or without adjuvant Most intramuscular delivery; 1 subcutaneous and several oral constructs	19	5
Replicating viral vectors	Avian paramyxovirus; horsepox; influenza; measles; Newcastle disease virus (NDV); vesicular stomatitis virus (VSV); Yellow fever virus (YF17D) Most intramuscular delivery; 2 intranasal	17	1
DNA	DNA plasmid vaccines; mostly S protein or RBD-based, but 2 also with N protein With or without adjuvant Most intramuscular delivery; some with electroporation/needle free delivery; 1 oral vaccine (bacTRL-Spike)	12	4
RNA	LNP-encapsulated encoding spike protein or RBD; self-amplifying Most intramuscular delivery; 1 intranasal	16	6
Virus like particle (VLP)	Whole virus, protein and peptides inside or on surface; lentivirus, baculovirus, HIV-based vehicles Some with adjuvants	12	1

*Adapted from WHO Draft Landscape of COVID-19 Vaccines [ref]*.

Several groups have developed mucosal SARS-CoV-2 vaccines. A deoptimized live attenuated whole virus vaccine is being developed for intranasal (i.n.) administration, and several adenovirus-based vaccines will also be administered via the i.n. route (Table 1). An oral probiotic pill-based SARS-CoV-2 DNA vaccine (bacTRL) is also planned for clinical trials (Table 1). The sublingual route is also considered an attractive route for inducing mucosal immunity (77). An alternative approach is to combine parenteral vaccines with adjuvants such as retinoic acid and CAF01 which are known to induce protective IgA mucosal responses (79, 80).

Researchers are also investigating self-administered mechanisms for SARS-CoV-2 vaccines to overcome the need for a healthcare worker to administer the vaccine. For example, Inovio^TM^ have developed a novel hand-held CELLECTRA® 2000 intradermal delivery device which uses a brief electrical pulse to reversibly open skin cell pores to allow the entry of their INO-4800 DNA plasmid vaccine (81) (Table 1). The University of Pittsburgh School of Medicine has developed an intradermal microneedle patch that can deliver SARS-CoV-2 protein antigens, either S or RBD (PittCoVacc), through the skin which will enter human trials soon (82) (Table 1). This approach delivers antigen and danger signals to the high-density dermal APCs stimulating a highly effective immune response, even in the absence of adjuvant. It requires only low doses of antigen thus reducing production costs. Indeed, this method induced more potent S1-specific nAbs in mice than the traditional needle injections, with or without the inclusion of TLR ligand sequences (83).

### Live Attenuated Vaccines

The development of live attenuated vaccines requires the organism to be modified via passage multiple times under unique conditions in the laboratory until it loses enough key virulent factors so as to not cause disease, but retains its ability to replicate in the vaccine recipient, and stimulate a robust immune response that protects against infection (84–86). These vaccines tend to be highly immunogenic, do not require an adjuvant and provide long-lasting immunity. However, they are usually contraindicated in those immunocompromised or pregnant.

It is difficult to develop a live attenuated SARS-CoV-2 vaccine quickly because of the time and knowledge required to ensure that it is suitably attenuated and all virulence factors removed. Reverse genetics can be employed for the rational design of the ideal attenuated SARS-CoV-2 vaccine virus, for example by determining key virulence factors, as has been done for other coronaviruses (87). Long-term maintenance of consistent live vaccine stocks is also problematic (85, 86). Despite the challenges, three codon-pair deoptimized live attenuated vaccines are currently undergoing pre-clinical testing, but none have yet progressed to clinical trials (7) (Table 1). These three candidates are being developed by Codegenix/Serum Institute of India, Indian Immunologicals Ltd/Griffith University and Mehment Ali Aydinlar University in Turkey, respectively (7). This approach relies on using a single virus strain which may not cross-protect against other strains, particularly as the virus continues to spread worldwide and mutates as selection pressure increases once immunity becomes more widespread.

### Inactivated Virus (Whole and Subunit) Vaccines

Inactivated vaccines are produced by growing the virus and then killing or “deactivating” it so that it can no longer replicate. The whole virus can be used, although alternative approaches include splitting the virus with a detergent to disrupt it or purifying antigenic components to create a subunit vaccine (88). These vaccines are safer than live-attenuated vaccines but less immunogenic and often require an adjuvant. There are currently four inactivated vaccines in human clinical trials and a further nine in the preclinical stages of development (Tables 1, 2). An alum adjuvanted purified formaldehyde inactivated whole virus vaccine developed by Sinovac Biotech., called PiCoVacc, has been shown to be immunogenic in mice, rats and non-human primates (96). It induced nAbs to both vaccine and non-vaccine strains and provided partial or complete protection of macaques in challenge studies and has now entered human clinical trials in China (Table 2). The China National Pharmaceutical Group (Sinopharm) have also developed inactivated whole virus vaccines with the Wuhan Institute of Biological Products and Beijing Institute of Biological Products which are currently in phase 3 clinical trials. The Wuhan vaccine has been shown to be safe and immunogenic in a randomized, double-blind, placebo-controlled phase 1/2 trial, which recently reported interim results (89). The phase 1 study tested three doses of 3 different vaccine concentrations (2.5, 5, and 10 μg per dose) of their alum adjuvanted vaccine vs. alum adjuvant alone placebo in 96 healthy adults; and the phase 2 tested two doses of 5 μg vs. alum alone in 224 18–59 year old healthy adults. Reasonable geometric mean anti-SARS-CoV-2 nAb titers (206–316) in 50% plaque reduction neutralization assays were induced at 14 days post-immunization. The vaccine did not appear to cause changes in serum innate (IL-1β, IL-6, IL-8, TNF-β), Th1 [IL-2, IL-12(p70), IFN-γ, TNF-α], Th2 (IL-4, IL-5, IL-10), or Th17 (IL-17, IL-21) cytokines, although formal statistical analysis of these data has not been reported. The safety data (Table 5) are discussed in section Comparative Safety and Tolerability Results for Current SARS-CoV-2 Vaccine Candidates in Clinical Trials. The Beijing inactivated vaccine candidate (BBIP-CorV) has published pre-clinical data confirming induction of high levels of nAbs in several animal models and protection against intra-tracheal SARS-CoV-2 challenge in rhesus macaques after 2 doses of vaccine (97). The Institute of Medical Biology/Chinese Academy of Medical Science has also developed an inactivated whole virus construct that is in a phase 1/2 trial; and Bharat Biotech International Ltd also has one in a phase 1/2 trial (Table 2).

**Table 2:** Candidate SARS-CoV-2 vaccines currently in clinical trials.

**Vaccine platform**	**Construct details**	**Route**	**Doses**	**Developer/Manufacturer**	**Clinical trial registration and stage**	**Age Gp (years)**
Inactivated whole virus	Formaldehyde inactivated with alum (PiCoVacc) Inactivated SARS-CoV-2 with alum Inactivated SARS-CoV-2 with alum (BBIP-CorV) Inactivated SARS-CoV-2 Whole virion inactivated (BBV152)	i.m. i.m. i.m. i.m. i.m.	2 2 2 2 2	Sinovac Biotech Wuhan Institute of Biological Products/Sinopharm Beijing Institute of Biological Products/Sinopharm Inst of Medical Biol/Chinese Acad Med Sci Bharat Biotech International Ltd	Phase 1/2 NCT04383574 Phase 1/2 NCT04352608 Phase 3 NCT04456595 Phase 1/2 ChiCTR2000031809 (89) Phase 3 ChiCTR2000034780 Phase 1/2 ChiCTR2000032459 Phase 3 ChiCTR2000034780 Phase 1 NCT04412538 Phase 1/2 NCT04470609 Phase 1/2 NCT04471519	≥60 18–59 ≥18 ≥6 ≥18 ≥6 ≥18 18–59 ≥60 12–65
Protein/peptide subunit	Adjuvanted recombinant RBD dimer S protein Full length S trimer (NVX-CoV2373) plus Matrix M^TM^ adjuvant RBD protein-based S protein Trimer-Tag© platform + ASO2 adjuvant (SCB-2019) Recombinant spike protein with Advax^TM^ adjuvant Molecular clamp stabilized S protein with MF59 adjuvant S-2P protein + CpG 1018 RBD protein subunit vaccine with adjuvant	i.m. i.m. i.m. i.m. i.m. i.m. i.m. i.m.	2 or 3 2 2 2 1 2 2 2	Anhui Zhifei Longcom Biopharmaceutical/Inst of Microbiol, Chinese Acad Sci Novavax Kentucky Bioprocessing, Inc. Clover Biopharmaceuticals Inc./GSK/Dynavax Vaxine Pty Ltd/Medytox Univ of Queensland/CSL/Seqiris Medigen Vaccine Biologics Corporation/NIAID/Dynavax Instituto Finlay de Vacunas, Cuba	Phase 1 NCT0445194 Phase 2 NCT04466085 Phase 1/2 NCT04368988 (90) Phase 1/2 NCT04473690 Phase 1 NCT04405908 Phase 1 NCT04453852 Phase 1 ACTRN12620000674932p Phase 1 NCT04487210 Phase 1/2 IFV/COR/04	18–59 18–59 18–59 18–70 18–75 18–65 18–55 20–50 19–80
Non-replicating viral vectors	Chimp adenovirus (ChAdOx1 nCoV-19) S protein (now called AZD1222) Adenovirus type 5 S protein (Ad5-nCoV) Adenovirus 26 (Ad26.COV2.S) Adenovirus 5 and 26 S protein (Gam-COVID-Vac) Replication defective Simian Adenovirus (GRAd) encoding S	i.m. i.m. i.m. i.m. i.m.	1 1 2 1 1	University of Oxford/Astra Zeneca CanSino Biological Inc./Beijing Institute of Biotechnology Janssen Pharmaceutical Gamaleya Research Inst. ReiThera/LEUKOCARE/Univercells	Phase 1/2 NCT04324606 (91) Phase 2 EUCTR 2020-001228-32 Phase 3 ISRCTN89951424 Phase 1 NCT04313127 (92) Phase 2 NCT04341389 (93) Phase 1/2 NCT04398147 Phase 1/2 NCT04436276 Phase 3 NCT04505722 Phase 1 NCT04436471 Phase 1 NCT04437875 Phase 1 EUCTR2020-002835-31	18–65 2–11, ≥18 18–55 18–60 ≥18 18–84 18–55, ≥65 ≥18 18–60 18–60 N/A
Replicating viral vectors	Measles virus vector-based (TMV-083)	i.m.	1 or 2	Institute Pasteur/Themis/Univ. of Pittsburg CVR/Merck Sharp & Dohme	Phase 1 NCT04497298	18–55
DNA	DNA plasmid vaccine S protein (INO-4800) CELLECTRA® electroporation device DNA plasmid vaccine DNA plasmid vaccine plus adjuvant (AG0301-COVID19) DNA vaccine (GX-19)	i.d. i.d. i.m. i.m.	2 3 2 2	Inovio Pharmaceuticals/International Vaccine Institute Cadila Healthcare Limited Osaka University/AnGes/Takara Bio Genexine Consortium	Phase 1/2 NCT04336410 Phase 1/2 NCT04447781 Phase 1/2 CTRI/2020/07/026352 Phase 1/2 NCT04463472 Phase 1/2 NCT04445389	≥18 19–64 18–55 20-65 18–50
RNA	LNP-encapsulated mRNA encoding stabilized S protein (mRNA-1273) LNP-encapsulated mRNA encoding trimerized RBD protein (BNT162) LNP-encapsulated mRNA (CVnCoV) LNP-encapsulated self-amplifying S protein mRNA (LNP-nCoVsaRNA)	i.m. i.m. i.m. i.m.	2 2 2 2	Moderna/NIAID BioNTech/Fosun Pharma/Pfizer CureVac Imperial College London	Phase 1 NCT04283461 (94) Phase 2 NCT04405076 Phase 3 NCT04470427 Phase 1/2 NCT04368728 (95) Phase 1/2 NCT04380701 Phase 3 NCT04368728 Phase 1 NCT04449276 Phase 1 ISRCTN17072692	18–99 ≥18 ≥18 ≥18 18–55 18–85 18–60 18–75
	mRNA mRNA (ARCT-021)	i.m. i.m.	2 2	People's Liberation Army (PLA) Academy of Military Sciences/Walvax Biotech. Arcturus/Duke-NUS	Phase 1 ChiCTR2000034112 Phase 1/2 NCT04480957	≥18 21–80
VLP	Plant derived VLP adjuvanted with AS03 or CpG 1018	i.m.	2	Medicago Inc.	Phase 1 NCT04450004	18–55
Immune cell therapy	DCs (LV-SMENP-DC) and Ag-specific CTL Artificial APCs Autologous DCs (AV-COVID-19) +/– GMCSF	s.c. i.v. s.c. s.c.		Shenzhen Geno-immune Medical Institute Shenzhen Geno-immune Medical Institute Aivita Biomed Inc.	Phase 1/2 NCT04276896 (in COVID-19 patients) Phase 1 NCT04299724 (in COVID-19 patients) Phase 1/2 NCT04386252	6m-80 6m-80 ≥18
Passive immunization	Convalescent plasma treatment Heat inactivated pooled plasma pill (V-SARS)	i.v. p.o.		Hilton Pharma Immunitor LLC	NCT04352751 (Phase not applicable) Phase 1/2 NCT04380532	18–55 18–65

*CTL, cytotoxic T lymphocyte; DC, dendritic cell; GMCSF, granulocyte-macrophage colony stimulation factor; LNP, lipid nanoparticle; NP, nanoparticle; S, spike; VLP, virus-like particle*.

### Protein and Peptide Vaccines

Rather than using the whole or a split fragment of the virus as the vaccine, antigenic proteins (or peptides) of the virus can be generated using recombinant technology in the laboratory instead (98). This is the most popular approach in the SARS-CoV-2 vaccine development field, with 50 protein/peptide constructs in preclinical trials and 8 that have entered clinical trials (Table 1). They are relatively simple vaccines to make and thus cheap to produce. A major advantage over whole virus vaccines is their relative safety since unnecessary reactogenic components such as lipopolysaccharides and toxins can be excluded. However, protein/peptide vaccines often require adjuvants and multiple doses in order to stimulate an adequate immune response. Peptide vaccines usually consist of 15–30 amino acid B cell and T cell epitope regions of key antigens allowing for a precise and targeted immune response. Peptide-based vaccines have been developed as candidates against several viral pathogens, but few have been licensed except for use in veterinary practice (99). This is in part due to their inherent limitations including a narrow breadth of immune response, potential for incorrect confirmation for epitope recognition, lack of B cell receptor cross-linking for B cell epitopes, HLA restriction for T cell recognition, failure to induce cross-reactive immunity against different viral strains and failure to elicit long-lasting immunity. The use of whole proteins in vaccines broadens their immunogenic potential, however the protein can also lose its native structure and thereby lose immunogenicity. In addition, as knowledge of the immune response to SARS-CoV-2 is still in its early stages, so too is the understanding of precisely what may be the most conformationally optimal, immunogenic and safe protein or peptide to utilize; this will be discovered as vaccine candidates using this approach are examined in clinical trials.

#### Focus on SARS-CoV-2 Spike Protein

The majority of SARS-CoV-2 candidate vaccines in development are based on the S protein or its RBD region (Figure 1) (100). Indeed, five of the current protein/peptide vaccine candidates in clinical trials target the full S protein and the other two target RBD only (Table 2).

Some novel technologies have been employed to overcome the issue of the S glycoprotein losing its immunogenic conformational form. The University of Queensland has developed a molecular clamp technique that uses proteins to stabilize the S protein in its coiled shape. This seeks to ensure that functional nAbs are produced that will tightly bind to virus in its native form and thus prevent cell entry (101) (Table 2). Other groups have focused on using powerful adjuvants to ensure immunogenicity, for example Novavax's recombinant S protein SARS-CoV-2 nanoparticle vaccine, NVX-CoV2373, combined with an adjuvant called Matrix-M1^TM^. This is a saponin-based adjuvant that enhances APC recruitment and action and stimulates high levels of nAbs and cell-mediated immune responses (102). The S protein in NVX-CoV2373 is present its native trimeric form and has been shown to stimulate high levels of nAbs in mice and baboons in preclinical studies. Primary phase 1 clinical trial results have just been reported from a randomized, observer-blind, placebo-controlled trial of NVX-CoV2373 conducted in 131 healthy adults (90) (Table 2). Safety and immunogenicity were assessed for 2 doses of vaccine (5 and 25 μg) with (*n* = 106) or without (*n* = 25) the Matrix-M1^TM^ adjuvant (a 50 μg dose). A strong correlation was observed between nAb titers and anti-spike IgG to S protein with the adjuvanted, but not the unadjuvanted, vaccine. Levels were comparable to those in convalescent sera (*r* = 0.958). Both 5 and 25 μg adjuvanted doses elicited responses of similar magnitude and every participant seroconverted by both assays. T cell responses measured in 16 participants showed that NVX-CoV2373/Matrix-M1 induces antigen-specific polyfunctional CD4^+^ T cell responses (IFN-γ, IL-2, and TNF-α) in response to spike protein stimulation; there was a strong bias toward this Th1 phenotype. The safety data are discussed in section Comparative Safety and Tolerability Results for Current SARS-CoV-2 Vaccine Candidates in Clinical Trials and this vaccine is now progressing to phase 2 trials.

Clover Biopharmaceuticals's protein Trimer Tag© vaccine is combined with AS02 adjuvant; the UQ vaccine uses MF59 adjuvant; the Vaxine Pty Ltd. S protein vaccine has a special Advax^TM^ adjuvant; and the Medigen S based construct is combined with the adjuvant CpG (Table 2). Generex Biotechnology, in partnership with Epivax, have developed a SARS-CoV-2 vaccine (EPV-Cov19) based on li-Key technology with undisclosed SARS-CoV-2 peptides (103). This uses synthetic peptides chemically linked to the 4-amino acid Ii-Key to ensure robust immune activation of Th1 and Th2 CD4^+^ T cells in particular, which in turn facilitate antibody production (104, 105).

### Viral Vector Vaccines

Multiple viral vectors have been used for vaccine delivery due to their ability to infect cells and deliver the gene product encoding antigenic proteins for production inside the host cell following administration, usually via the intramuscular (i.m.) route (106). Vector viruses are usually genetically modified to reduce their virulence and render them replication defective. Adenoviruses and poxviruses are the most widely used non-replicating viral vectors, but many other viruses can also be adapted for vaccine delivery. Replicating viral vectors, such as measles virus and vesicular stomatitis virus (VSV), can also be used for SARS-CoV-2 vaccine design; they mimic natural infection, rendering them self-adjuvanting and therefore more potent, and can be used in lower doses (107). While subunit vaccines are more focused on induction of humoral immunity, viral vector vaccines are able to induce robust cell mediated immunity (CMI) in addition to Abs and have been shown in animal models to protect against challenge with pathogenic coronaviruses (51). A modified vaccinia Ankara (MVA) vector expressing a MERS-CoV recombinant protein induced T cell responses and nAbs in mice, while a rabies virus-based SARS-CoV-1 vaccine protected mice against SARS-CoV-1 challenge (13).

#### Non-replicating Viral Vector SARS-CoV-2 Vaccines

Human adenoviruses are the most common non-replicating viral vectors being used for SARS-CoV-2 vaccine development; constituting 3 of the 5 in clinical trials and 7 of the 19 in preclinical development (Table 1). The three in clinical trials include CanSino's Ad5-nCoV, Janssen's Ad26COVS1 (108) and Gamelaya's Ad26-based Gam-COVID-Vac Lyo vaccine (Table 2). All three encode the S protein. While adenovirus vectors are well-tolerated and highly immunogenic in most people, pre-existing immunity to the viral vector can hamper the induction of immunity, particularly after repeated doses. Animal adenoviruses can be used as vectors instead of human ones to overcome this problem, which is the approach used for the Oxford University chimpanzee adenovirus-based candidate SARS-CoV-2 vaccine ChAdOx1 nCoV-19 (also called AZD1222) and the simian adenovirus-based ReiThera vaccine GRAd, both of which are in clinical trials (Table 2). Anti-vector immunity can still develop after the first dose of vaccine even if a simian adenovirus vector is used. Despite the concern about anti-vector immunity, 4 of the 5 adenovirus-based SARS-CoV-2 vaccines in clinical trials are planned to be given as a single dose, whereas almost all the other vaccines in clinical trials require at least 2 doses (Table 2).

Phase 1 and 2 trials have now been completed and published for the Cansino (Ad5-nCoV) and Oxford (ChAdOx1 nCoV-19), the safety aspects of which will be discussed later in this article. The Ad5-nCoV randomized, double-blind, placebo-controlled phase 2 trial results involving 603 volunteers ≥18 years of age showed >95% seroconversion by RBD-ELISA, but only 59% seroconversion of nAb responses in the higher dose group and 47% in the lower dose group after a single immunization (93). Fifty two percent of volunteers had pre-existing anti-Ad5 nAbs, and those with higher anti-Ad5 immunity had approximately half the RBD-specific ELISA and SARS-CoV-2 nAbs as compared to those with low pre-existing anti-vector immunity, supporting interference with vaccine immunogenicity. Preliminary findings from a phase 1/2, single-blind, randomized controlled ChAdOx1 nCoV-19 vaccine trial conducted in 1,077 healthy volunteers aged 18–55 years induced nAbs in 91% of participants after a single dose of vaccine, rising to 100% after a booster dose (91). Neutralization titers were comparable to those observed in convalescent plasma from people who had recovered from COVID-19.

Both vaccine constructs elicited T cell responses which peaked at 14 days post-immunization, as determined by interferon-gamma (IFN-γ) enzyme linked immunospot (ELISpot) assay (91, 92). Day 14 responses appeared higher with ChAdOx1 nCoV-19 (median 856 per million peripheral blood mononuclear cells [PBMC]) as compared to the highest dose used of Ad5-nCoV (mean 580 per million PBMC) in the phase 1 trial (92); Ad5-nCoV elicited a median of 100–110 SFU/million PBMC at day 28 in the phase 2 trial (93). Intracellular staining confirmed production of IFN-γ, tumor necrosis factor (TNF) and interleukin-2 (IL-2) by both CD4^+^ and CD8^+^ T cells following Ad5-nCoV immunization, with more cytokine detected in CD4^+^ T cells as compared to CD8^+^ T cells (92).

#### Replicating Viral Vector SARS-CoV-2 Vaccines

Institute Pasteur replicating viral vector vaccine candidate TMV-083, based on measles virus, is the only viral vector vaccine that has entered phase 1 clinical trials to date (Table 2), but there are 17 more at the pre-clinical stage of development. These include 5 each based on influenza virus or VSV, 3 more measles virus-based candidates, and 1 each based on avian paramyxovirus, horsepox (called TNX-1800), Newcastle disease virus, and yellow fever virus (Table 1). The majority target the S protein or RBD, although one measles virus-based candidate also targets the N protein. Two of the influenza-based candidates are designed for intranasal administration. A live attenuated yellow fever vector-based vaccine (YFS0) expressing the prefusion form of the S protein recently showed that a single dose protected most hamsters in a SARS-CoV-2 challenge model (109).

### Nucleic Acid-Based Vaccines (DNA or mRNA)

Nucleic acid-based vaccines (DNA or mRNA) offer a cost-efficient and scalable approach to SARS-CoV-2 vaccine development (110). The major nucleic acid-based approaches are described below.

#### DNA Vaccines

DNA-based vaccines are stable and safe to handle. However, while there are multiple DNA vaccine constructs targeting numerous viral infections in animal and human studies, to date none have been licensed for human use. Naked DNA can be injected and taken up by APC or APC can be directly transfected with plasmid DNA encoding the target antigen. Subsequent expression and presentation of the target antigen leads to the induction of antigen-specific CD4^+^ and CD8^+^ T cells and antibody production. However, DNA vaccines tend to be poorly immunogenic, necessitating various strategies to improve immunogenicity such as the use of viral promotors, immunostimulatory sequences and adjuvants. Nanocarriers such a virus like particles (VLPs) can also be used to improve DNA delivery, avoid its degradation and be immunostimulatory. There are safety concerns about potential integration into the host DNA causing dysregulated gene expression although the risk of this is thought to be very low.

A series of animal studies with SARS-CoV-1 nucleic acid-based vaccines have shown induction of nAbs and cellular immunity, with partial protection against turkey CoV challenge (13) and vaccine-induced Ab-mediated protection to the S protein in mice (51). Twelve DNA-based SARS-CoV-2 vaccines are currently in preclinical development and 4 more have entered phase 1/2 clinical trials (Table 1). Two DNA-based candidates in clinical trials are administered i.m., whereas the other 2 are administered via the intradermal (i.d.) route (Table 2). Inovio Pharmaceuticals is testing their CELLECTRA® electroporation i.d. delivery device to administer their S protein-based DNA plasmid vaccine INO-4800 which induces nAbs and T cells in mice and guinea pigs (111) (Table 2). An alternative approach currently in pre-clinical trials is being taken by Symvivo with their DNA *B. longum* vaccine bacTRL which is administered as an oral pill (112).

#### Messenger RNA-Based Vaccines

Messenger RNA (mRNA) vaccines consist of an RNA strand that codes for a target antigen (113, 114). The two main types are those packaged and delivered in non-replicating form or those packaged with other RNA as an *in vivo* replicating vaccine. They offer a promising alternative to conventional vaccine approaches due to their high potency, capacity for rapid development and potential for low-cost manufacture, which could prove crucial for global accessibility for a COVID19 pandemic vaccine. They also have a good safety profile, since they are not made with pathogen and do not integrate into host DNA. Several challenges encountered by mRNA vaccines include the need for packaging, for example into particles or liposomes, as naked RNA is otherwise rapidly broken down and the need for freezing or refrigeration for wide scale delivery.

There are currently 16 RNA-based vaccines in pre-clinical trials and 6 have entered clinical trials (Table 1). The clinical trial candidates include two lipid nanoparticle encapsulated RNA SARS-CoV-2 vaccines that have already progressed to phase 3 trials (Table 2). Moderna/NIAID have published preliminary results from their phase 1 dose-ranging, safety and immunogenicity trial of 2 doses of mRNA-1273 encoding the S protein conducted in 45 healthy adults aged 18–55 years (94). The S protein in mRNA-1273 is stabilized in its prefusion state by 2 proline substitutions in the S2 subunit; and the lipid nanoparticle coat consists of 4 lipids. All participants developed RBD Abs by ELISA and SARS-CoV-2-specific nAbs by neutralization assay after 2 doses in the upper range of those who have recovered from COVID-19 (convalescent serum). T cell responses measured by intracellular staining for Th1 (TNF, IL-2, IFN-γ) and Th2 (IL-4, IL-13) cytokines following stimulation with an S protein peptide pool showed a Th1 cytokine bias and low-level CD8 T cell responses. An interim report from the Phase 1/2 dose-ranging study of 2 doses of BioNTech's mRNA S protein vaccine candidate BNT162 administered to adults aged 18–55 years elicited RBD-binding IgG and SARS-CoV-2 nAbs in the order of 1.8–2.8-fold that of convalescent human plasma. T cell responses were not reported in this paper. This vaccine encodes trimerized RBD which is modified by adding a “foldon” trimerization domain to increase immunogenicity (114). Both mRNA-1273 and BNT162 are now in phase 3 clinical trials in adults ≥18 years (Table 2). The safety data for both will be reviewed in a later safety section.

The remaining RNA-based candidates in clinical trials include three in phase 1 and one in phase 1/2 (Table 2). The Imperial College London LNP-encapsulated RNA vaccine consists of an S protein-based self-amplifying RNA construct (LNP-nCoVsaRNA); designed because saRNA vaccines induce a more stable DNA product and are more immunogenic than conventional nucleic acid vaccines (115) (Table 1).

### Nanocarriers and Virus-Like Particle Vaccines

Nanoparticle-based vaccine approaches have received increasing interest in recent years due to their good safety profile and high immunogenic potential, with an ability to efficiently target dendritic cells (DCs) for processing and presentation, providing a clear advantage over less immunogenic DNA and RNA vaccines (116). Materials used to construct nanoparticle vaccines include self-assembling viruses (virus like particles [VLPs]), lipids (as liposomes), proteins, metals (e.g., gold) and polymers which also act as their own adjuvant (117).

Many nanoparticles are highly stable and less prone to degradation than other constructs such as “naked” protein, peptide, DNA and RNA vaccines (116, 118). Selected antigens, which may be proteins or nucleic acid, can either be attached to the surface of the nanoparticles or combined in the particle core. They can be synthesized in a variety of shapes and sizes to induce robust innate as well as adaptive immunity. Other modifications include altering the nanoparticle surface to target certain cells or enhance immunogenicity and packaging with TLR ligands and other immune modulators.

One of the most popular approaches for viral vaccine development is engineering VLPs consisting of self-assembled viral membrane in a monomeric complex which display the viral epitopes but lack multiple key viral components, ensuring they have no replication capacity (119). The widely used human papillomavirus (HPV) vaccines are an example of this approach. A nanoparticle polypeptide vaccine displaying SARS-specific B cell epitope repeats from the C terminal heptad repeat region of the S protein in its native coiled formation was shown to elicit nAbs in mice (120).

A number of the protein and nucleic acid-based SARS-CoV-2 vaccine candidates use nanocarriers to package the vaccine product to improve stability and ensure efficient antigen processing (118). As mentioned above, Novavax's full length S protein construct NVX-CoV2373 is a nanoparticle vaccine and the majority of mRNA-based SARS-CoV-2 vaccines are packaged LNPs, including mRNA-1273 and BNT162. There are 12 SARS-CoV-2 VLP vaccines in preclinical development and just one in a phase 1 clinical trial (Table 1). The latter vaccine developed by Mendicago Inc. consists of plant-derived recombinant VLPs made by transfecting viral genes into the cell nuclei of leaves permitting transient expression of viral proteins which form into VLPs which are extracted and purified for clinical use (121) (Table 2).

### Immune Cell Therapy

SARS-CoV-2 vaccine approaches based on CAR-T cell concepts are also being tested in clinical trials. The Shenzhen Geno-immune Medical Institute in China is using a lentiviral vector system to create viral minigenes which express viral proteins (S, M, E and N proteins) and immune modulatory genes (P polyprotein protease) to modify dendritic cells (DCs). The LV-DC presenting SARS-CoV-2 specific antigens will activate cytotoxic T cells. Novel DC (LV-SMENP DC) and antigen-specific cytotoxic T cell vaccines are currently being tested by s.c. injection or i.v. infusion in a phase 1/2 multicenter therapeutic clinical trial in participants ranging from 6 months to 80 years of age (122) (Table 2). The same company has also developed artificial DCs expressing SARS-CoV-2 mini-genes for various viral proteins which are being tested in a phase 1 clinical trial as a s.c. injection in SARS-CoV-2 infected individuals in the same age range (Table 2). Autologous DCs expressing SARS-CoV-2 antigens have also been developed by Aivita Biomed Inc. in USA and are being tested as a s.c. administered vaccine in phase 1 clinical trials in healthy adults >18 years of age (123) (Table 2). Whilst not suitable for large-scale delivery and use, immune cell therapy might be used in the context of pre-emptive therapy in high-risk patients such as the elderly and immunocompromised.

## Repurposing Old Vaccines to Provide Non-Targeted Antiviral Immunity

There is increasing evidence that immunization with live vaccines can improve survival against non-vaccine targeted infections, a phenomenon termed “non-specific,” “off-target,” or “heterologous” effects of vaccines (124, 125). Some of the best evidence comes from studies of immunization with the tuberculosis vaccine bacillus Calmette-Guérin (BCG); for example, BCG has been shown in three randomized trials to markedly reduce all-cause mortality in low birthweight neonates (126). BCG causes epigenetic modification of innate immune cells (monocytes and natural killer (NK) cells) thereby enhancing innate responses in a process called innate immune training (127, 128). Experiments in both murine models and humans have further shown that BCG protects against certain viral pathogens (129).

Reports that people living in countries where childhood BCG is routinely given have lower COVID-19 mortality have been posited as further evidence for protective effects of BCG (130). However, these observations are fraught with confounders, including difficulty ascertaining COVID-19 infection rates in these countries, the time the virus first entered the country and differences in national control strategies (131). Furthermore, other studies contradict these findings, such as a geographic regression discontinuity analysis along the East and West German border showing that the COVID-19 infection rates do not vary according to the BCG vaccination strategies employed during the cold war (132); and a study from Israel showing that SARS-CoV-2 infection rates in adults did not differ by BCG at birth status (133).

Even if childhood BCG immunization does not protect against COVID-19, a BCG dose as an adult may provide some short-term protection. Indeed, it has been widely postulated that the non-specific beneficial effects of BCG might protect against COVID-19, resulting in an explosion of interest in this vaccine as a protective strategy in recent months (134, 135). This has led to a number of clinical trials being established in North and South America, Australasia, Africa and Europe, all testing the ability of BCG vaccine to protect against SARS-CoV-2 infection, mostly in high-risk exposed healthcare workers (136) (Table 2). These trials collectively aim to recruit >20,000 participants, with the largest trial of 10,078 healthcare workers currently recruiting across multiple sites in Australia and Europe.

Non-specific protective effects of live vaccines have also been postulated for oral polio vaccine (OPV) (137) and measles vaccine (138). As a result, a study of the protective effects of OPV against COVID-19 is due to commence in USA in June (139), and a clinical trial of measles-mumps-rubella (MMR) immunization and COVID-19 protection is being conducted in Egypt (Table 3).

**Table 3:** Clinical trials of BCG and MMR immunization for protection against COVID-19 (from clinicaltrials.gov).

**Vaccine**	**Target population**	**Age (yrs)**	***N***	**Primary endpoint**	**Sponsor**	**Country**	**Clinical trial identifier**
BCG	Healthy HCWs in ED, ICU, isolation ward	≥18	900	Incidence of COVID-19 cases	Ain Shams University	Egypt	NCT04350931
BCG	COVID-19 patients TST+ and TST- Case-Control	12–80	100	Pneumonia severity, need for ICU admission in TST+ and TST- patients	Assiut University	Egypt	NCT04347876
BCG	COVID-19 patients	≥18	1,000	COVID-19 progression, elimination, seroconversion	University of Campinas	Brazil	NCT04369794
BCG	COVID+ patients or COVID- patients who have been in contact with COVID-19	18–80	400	Differences in epidemiological characteristics	Direction des Soins de Santé de Base	Tunisia	NCT04384614
BCG	Healthy HCWs treating COVID-19 patients	18–100	1,500	HCW absenteeism	Bandim Health Project	Denmark	NCT04373291
BCG	Healthcare workers	≥18	1,500	HCW absenteeism	UMC Utrecht	Netherlands	NCT04328441
BCG	Healthy HCWs in contact with COVID-19 patients	≥18	500	Incidence hospitalized due to COVID-19	TASK Applied Science	South Africa	NCT04379336
BCG	Healthy HCWs treating COVID-19 patients	18–65	1,000	Incidence of COVID-19 cases	University of Antioquia	Columbia	NCT04362124
BCG	Healthy HCWs (BRACE Trial)	≥18	10,078	COVID-19 disease incidence and severe COVID-19 incidence	Murdoch Children's Research Unit	Australia	NCT04327206
BCG	Healthy HCWs in direct contact with COVID-19 patients (≥25 h/wk)	18–75	1,800	Incidence of COVID-19 infection	Texas A&M University	USA	NCT04348370
BCG	Healthy HCWs in contact with COVID-19 patients	≥18	1,120	Incidence of COVID-19 infection	Assistance Publique—Hôpitaux de Paris	France	NCT04384549
MMR	Healthy HCWs during COVID-19 outbreak	18–50	200	Incidence of COVID-19 disease	Kasr El Aini Hospital	Egypt	NCT04357028

*BCG, bacillus Calmette-Guérin vaccine; MMR, measles-mumps-rubella vaccine; HCW, healthcare worker; TST, tuberculin skin test*.

A consistent feature of the off-target effects of live vaccines has been their sex-differential nature; females often showing greater susceptibility to these immunomodulatory effects (124, 125, 140–142). Indeed, sex differences in targeted vaccine immunogenicity and adverse events have also been widely described (141, 142). This raises the possibility that any off-target protective effect of live vaccines may differ between the sexes and that the SARS-CoV-2 vaccine candidates may show sex differences in immunogenicity and reactogenicity.

It is possible, but uncertain, that strategies to use these already licensed vaccines may provide a modest level of protection against COVID-19, including those at highest risk, such as healthcare workers. It is important to recognize, however, that data from these studies will still need to accrue whilst we are waiting for SARS-CoV-2 targeted vaccines to come through the pipeline. Given the unprecedented speed at which targeted vaccines are being developed, this approach may not be necessary or required; furthermore, these existing live vaccines are not currently recommended for this indication, so should only be utilized in this context in the setting of a clinical trial.

## Potential Challenges in SARS-CoV-2 Vaccine Development

Even if an efficacious vaccine is developed there are a number of immunological challenges that need to be considered. Furthermore, there is the enormous challenge of mass production and rapid and equitable delivery (Table 4).

**Table 4:** Some of the key challenges to successful SARS-CoV-2 vaccine development.

**Challenges to SARS-CoV-2 Vaccine Development**
Induction of only modest protection
Aberrant Ab responses: OAS and ADE
Aberrant T cell responses: VAERD, Th2 skewing, OAS
Vaccine AEs, SAEs, AESI
Determining efficacy in humans
Lack of standardized assays for measuring Abs and CMI
High development costs
Logistics of mass production
World-wide delivery and vaccine program implementation
Affordability for poorer nations
Long-term sustainability if regular doses needed

*ADE, antibody dependent enhancement; AE, adverse event; AESI, adverse event of special interest; OAS, original antigenic sin; SAE, serious adverse event; VAERD, vaccine associated enhanced respiratory disease*.

### Potential for Modest Vaccine Protection

It is extremely unlikely that any of the SARS-CoV-2 vaccines will be 100% effective; while they may not prevent becoming infected, it is hoped that they will prevent progression to severe disease. Indeed, the seasonal influenza vaccine is generally about 50% protective against infection but does decrease disease severity and hospitalization rates (143). A recent study in which macaques were vaccinated with the Oxford University and AstraZeneca adenovirus vaccine, ChAdOx1 nCoV-19, found that the primates were protected from SARS-CoV-2-induced pneumonia (144). However, the macaques still had high levels of virus replicating in their upper respiratory tract. It is hoped that even if the vaccines do not prevent infection in the upper airways, they may reduce viral load and disease severity and in turn, the amount of virus a vaccinated person transmits to others. Even a modestly efficacious SARS-CoV-2 vaccine could mitigate the severity of this pandemic and be highly beneficial in a world struggling to contain this novel virus and its devastating social and economic effects.

Most vaccines are tested in healthy young adult males and non-pregnant women and, if safe, they are then tested in healthy children prior to licensure. This therefore raises the issue that any vaccines may initially have less empiric data available on use in certain key vulnerable populations such as the elderly, immunocompromised groups and pregnant women. It is plausible that vaccines may be considerably less immunogenic in older and frail elderly who experience the most severe outcomes from COVID-19, hence the importance of adjuvants in many of the vaccine candidates, both for dose sparing and enhancing immunogenicity (145). Efficacy in this population is also likely to vary by the type of vaccine construct and adjuvant used. This has been seen for other diseases such as influenza, where the adjuvanted and high dose vaccines are more immunogenic than the standard inactivated vaccines in the elderly (146, 147). Similarly, for herpes zoster, the efficacy of the live-attenuated vaccine in the elderly is approximately 60% compared with >90% for a subunit adjuvanted vaccine (glycoprotein E and liposome-based AS01B adjuvant) (148). Thus, ensuring studies can be conducted in these priority target groups, either pre- or early-post deployment, will be critical and special formulations may be required to ensure adequate immunogenicity.

### Cross-Reactive Coronaviruses Antibodies, Original Antigenic Sin and Antibody Dependent Enhancement (ADE) of Immunity

Throughout life, humans are repeatedly exposed to the endemic human seasonal coronaviruses and develop human CoV (hCoV) antibody repertoires that can potentially cross-react with SARS-CoV-2-specific antigens (149). This early immune imprinting leads to preferential expansion of cross-reactive Abs when a related antigen is encountered, in this case SARS-CoV-2 (150). This long-recognized process is called original antigenic sin (OAS) (151) and is known to occur with several common viruses, such as influenza and respiratory syncytial virus (152, 153). OAS can either lead to a less effective immune response or cause enhanced immunity and immunopathology (149, 150, 154). OAS is one of the mechanisms responsible for the phenomenon of antibody dependent enhancement (ADE) of immunity which is thought to occur in COVID-19, whereby non-neutralizing cross-reactive Abs against other coronaviruses enhance host cell entry and viral infectivity and worsen disease severity (150, 154, 155). This was seen to occur in cats vaccinated against feline infectious peritonitis coronavirus (156–158) and certain SARS and MERS vaccine platforms also appear to have shown worsened immunopathology in animal challenge studies compared with placebo (159).

Recently, it was shown *in vitro* using human cells that nAbs to coronavirus S protein can also trigger ADE by causing a conformational change of the S protein (160). Antigen-experienced older individuals are more susceptible to the phenomenon of ADE, while less antigen-experienced younger individuals mount more targeted antibody responses to viral neoantigens. This may in part account for the greater immunopathology of COVID-19 in older individuals. Indeed, it has been shown recently that COVID-19-naïve children and elderly have quite different cross-reactive SARS-CoV-2-specific antibody signatures, which would play differing roles upon challenge with the wild-type virus (161).

Theoretically, a SARS-CoV-2 vaccine could skew the immune system toward production of less effective or even harmful cross-reactive hCoV Abs via these processes of OAS and ADE, rather than induce highly-targeted SARS-CoV-2-specific Abs as intended (162). Indeed, anti-spike Abs taken from critically unwell COVID-19 patients with severe lung injury skewed macrophages *in vitro* into a pro-inflammatory phenotype, implicating the Abs in driving the surge in lung-resident pro-inflammatory macrophages and subsequent pro-inflammatory cytokine release in the lungs (163). The authors attribute an aberrant IgG glycosylation pattern in severe COVID-19 patients with their more pro-inflammatory properties so hopefully the IgGs induced by vaccination will not have this problem. Indeed, none of the current SARS-CoV-2 vaccines in human clinical trials have reportedly caused ADE to date, although the number of human subjects studied to date is still relatively small. Since animal challenge data are not required to progress to clinical SARS-CoV-2 vaccine trials, the opportunity to screen for potential adverse events including ADE at the pre-clinical stage of development may be missed. Having said that, many of the SARS-CoV-2 vaccine candidates have undergone considerable pre-clinical testing, including challenge studies. There was no evidence of ADE in pre-clinical SARS-CoV-2 challenge studies in rhesus macaques following immunization with 1 dose of ChAdOx1 nCoV-19 (144) or 2 doses of the inactivated whole virus vaccine candidate BBIBP-CorV (97), both of which were protective in these studies. It is postulated that basing the vaccine on the S1 RBD terminal subunit of the S glycoprotein should overcome this problem by inducing nAbs only, although this has not been proven and, notably, only a few vaccine candidates have taken this approach. Furthermore, the *in vitro* conformational changes causing ADE described above occur in the RBD domain, so this approach may not be successful.

### Aberrant T Cell Responses

Aberrant manifestations of T cell responses were observed in the 1960s for other viral vaccine candidates, such as inactivated vaccines against measles and respiratory syncytial virus (RSV), resulting in vaccine associated enhanced respiratory disease (VAERD) upon subsequent wild-type pathogen exposure (164, 165). A major driver of this was accentuation of Th2 cytokines, with resultant allergic (eosinophilic) and airway hyperesponsiveness. This was compounded by another mechanism related to the conformation of the vaccine antigen, resulting in the generation of excessive non-neutralizing Ab. Having a high amount of binding, but not neutralizing, Ab caused immune complex deposition and complement activation with local inflammation in the presence of a high viral load. It will therefore be important for vaccine candidates to mitigate the risk of VAERD by considering the T cell profile induced by vaccination, avoiding Th2 biased CD4^+^ T cell immunity and biasing toward CD8^+^ cytotoxic T cell responses. The alum adjuvanted inactivated vaccine candidate PiCoVacc could theoretically drive a Th2 skewed immune response. While this was not observed in macaque studies when analyzing Th1 and Th2 cytokine profiles, it is not clear from the paper what samples were used to measure these cytokines, clarification of which will be crucial in vaccine safety assessment (96). Many of the other vaccine constructs favor Th1 immunity, as demonstrated in the published human trial results for several of the viral vector (91–93) and mRNA vaccines (94) and the Novavax nanoparticle Matrix M adjuvant vaccine (90). Original antigenic sin also applies to T cell responses and therefore pre-existing CoV-specific T cell memory may be enhanced by immunization with a SARS-CoV-2 vaccine leading to either suboptimal T cell responses or immunopathology (151, 166).

### Vaccine Safety Assessment

#### Vaccine Safety Monitoring Pre and Post-deployment

Vaccine safety assessment is integrated into all stages of the clinical development pipeline for candidate vaccines and continues once vaccines are deployed into the population. Randomized controlled clinical trials examine safety, as well as immunogenicity and efficacy. Decisions on the participant numbers to be included in phase 3 studies will likely be powered on efficacy endpoints, but these studies also need to be of sufficient size and planned duration to ensure comparison of injection site, systemic and unanticipated or “unsolicited” adverse events between groups. Comprehensive safety studies are particularly critical because some candidate vaccines use platform technologies that have not been examined extensively in human subjects to date, including some of the viral vectors, mRNA and nanoparticle constructs, and because of the potential for enhanced disease and adverse events related to aberrant immune responses to be seen upon infection pre- and post-licensure. A list of adverse events of special interest (known as AESI) across all body systems, including immunological, cardiovascular, neurological, musculoskeletal and dermatological manifestations, have been agreed by the Brighton Collaboration in conjunction with CEPI and the WHO with input from regulatory agencies and other experts, as have the associated case definitions and surveillance strategies (167, 168). Listed conditions include anaphylaxis, vasculitides, myocarditis, generalized convulsions and meningoencephalitis, among many others. Comprehensive data on safety are essential to ensure that the benefit:risk ratio of vaccination is favorable, to support decision-making by policy makers on wide-scale program implementation among healthy people, and to ensure that individuals are sufficiently confident to accept vaccination. Some of these AESI will require post marketing (phase 4) studies but are also undergoing evaluation in some of the pre-clinical and early phase evaluations, particularly the larger phase 3 studies.

#### Comparative Safety and Tolerability Results for Current SARS-CoV-2 Vaccine Candidates in Clinical Trials

Interim phase 1/2 safety results have recently been published for two leading adenovirus-based vaccines, ChAdOx1 nCoV-19 (91) and Ad5-nCoV (92, 93); two mRNA based vaccine candidates, mRNA-1273 (94) and BNT162 (95); an aluminum adjuvanted inactivated whole virus vaccine (89); and an adjuvanted recombinant protein nanoparticle vaccine, NVX-CoV2373/Matrix-M1 (90). All candidates exhibited acceptable safety profiles, and while local and systemic reactogenicity was common for the mRNA, adenovirus and adjuvanted protein nanoparticle constructs, the inactivated whole virus vaccine was considerably less reactogenic (Table 5). Reactions were generally mild to moderate and resolved within days. Importantly, there were no serious adverse events reported for any of these vaccine candidates.

**Table 5:** Summary of phase 1/2 safety reporting results for ChAdOx1 nCoV-19, Ad5-nCoV, mRNA-1273, and BNT-162.

**Vaccine and dose**	**Number tested**	**Pain (%)**	**Fatigue (%)**	**Headache (%)**	**Myalgia (%)**	**Fever ≥38°C (%)**	**Serious adverse event**
**ChAdOx1 nCoV-19 (phase 1/2) (****91****)**							
1 dose 5 × 10^10^ v.p. (no PCM)	487	67	70	68	60	18	0
1 dose 5 × 10^10^ v.p. (with PCM)	56	50	71	61	48	16	0
**Ad-5 nCoV (phase 1) (****92****)**							
1 dose 5 × 10^10^ v.p.	36	47	47	39	19	42	0
1 dose 1 × 10^11^ v.p.	36	56	39	31	8	42	0
1 dose 1.5 × 10^11^ v.p.	36	58	44	47	22	56	0
**Ad-5 nCoV (phase 2) (****93****)**							
1 dose 5 × 10^10^ v.p.	129	56	34	28	18	16	0
1 dose 1 × 10^11^ v.p.	253	57	42	29	15	32	0
**mRNA-1273 (phase 1) (****94****)**							
2 doses 25 μg	13	77	39	23	23	0	0
2 doses 100 μg	15	100	80	60	53	40	0
2 doses 250 μg	14	100	57	93	86	57	0
**BNT162b1 (phase 1) (****95****)**							
2 doses 10 μg	12	83	66	83	41	8.3	0
2 doses 30 μg	12	100	83	100	59	75	0
1 dose 100 μg	12	100	84	75	58	50	0
**Whole inactivated alum adjuvanted vaccine (phase 1) (****89****)**							
3 doses 2.5 μg	24	21	0	0	0	0	0
3 doses 5 μg	24	4	4	0	0	4	0
3 doses 10 μg	24	25	0	0	0	4	0
**Whole inactivated alum adjuvanted vaccine (phase 2) (****89****)**							
2 doses 5 μg day 0 and 14	84	2	1	1	0	5	0
2 doses 5 μg day 0 and 21	84	14	0	0	0	2	0
**NVX-CoV2373/Matrix-M1 (phase 1) (****90****)**							
2 doses 5 μg + 50 μg Matrix-M1	25	58	47	47	45	0	0
2 doses 25 μg + 50 μg Matrix-M1	25	62	50	58	55	4	0
2 doses 25 μg no adjuvant	25	8	12	28	9	0	0

*Results for the 2 or 3 dose schedules show reported results after the final dose. Values approximated where data presented as figures. PCM, paracetamol; v.p., viral particles*.

Pain at the injection site was a particularly common feature with the mRNA vaccines (approaching 100% in some groups), and while it was the commonest reaction to the inactivated vaccine, it was still lower for this candidate than for the other vaccines (Table 5). Headache, myalgia and fatigue were the other most commonly reported symptoms for the mRNA, adenovirus and adjuvanted protein nanoparticle candidate vaccines (Table 5). Documented fever was relatively common for mRNA and adenovirus candidates but ≤5% for the inactivated virus and adjuvanted protein nanoparticle candidates (Table 5). The ChAdOx1 nCoV-19 trial underwent a protocol amendment during the trial allowing several sites to administer paracetamol prior to vaccination and 6 hourly for 24 h. This slightly reduced reports of pain, feeling feverish, chills, mylagia, headache and malaise but had no effect on confirmed fever. Pre-existing adenovirus 5 immunity, increased age and male sex were all associated with decreased fever following immunization with Ad5-nCoV (93). In terms of blood parameters, immunization with ChAdOx1 nCoV-19 was associated with transient neutropaenia and BNT162b1 with lymphopaenia.

In the Ad5-nCoV phase 1 and 2 trials reactogenicity was dose-dependent for pain, headache and fever; while it was dose-dependent for almost all parameters for the mRNA vaccines (Table 5). As a result of the high reactogenicity after a single dose of BNT162b1, it was decided not to give a second dose. Interestingly, the ten participants given a booster dose of the ChAdOx1 nCoV-19 vaccine had less reactogenicity after the second dose. The opposite was found for the BNT-162b1 mRNA and NVX-CoV2373/Matrix-M1vaccines for which reactogenicity was greater after the second dose (90, 95).

These favorable safety results (alongside the good immunogenicity reported earlier in this article) have allowed the progression of the adenovirus and mRNA candidate vaccines to phase 3 clinical trials.

### Testing Vaccine Efficacy and Human SARS-CoV-2 Challenge Trials

Whilst the SARS-CoV-2 vaccine pipeline is progressing at unprecedented speed, there is a concern that suppression of viral transmission in many countries will make evaluation of vaccine efficacy (VE) difficult, as phase 3 studies need sufficient infection rates to compare disease incidence in vaccinated with control individuals. Reassuringly, planned studies are currently expanding to higher burden countries where infrastructure to conduct complex adaptive clinical trials exists. Nevertheless, the time needed to accrue sufficient data on efficacy will be a critical determinant impacting vaccine availability. In the setting of this uncertainty “human challenge models” for SARS-CoV-2 have been proposed. This methodology involves the intentional infection of research participants, providing safety data and pointing to immune correlates of protection. This approach has recently advanced vaccine development for infections like *Salmonella typhi* (typhoid vaccine) and Group A Streptococcus (169, 170). The difference between these bacterial human challenge models and SARS-CoV-2 is that these pathogens have been researched for decades and have an effective antibiotic “rescue” treatment available. Infecting a human with SARS-CoV-2 to test vaccine efficacy raises numerous ethical issues (171), including those around informed consent of the healthy volunteer, noting that while severe COVID-19 is less common in young adults, deaths have still occurred in this age group. Strict infection control and personal protective equipment (PPE) measures would also be required to limit “third-party” risk to staff co-ordinating any studies. While it is uncertain if this approach will be required, the WHO is progressing a framework, including key criteria, that will be required by ethical review boards in order to facilitate “human challenge” trials (172). As noted by Stanley Plotkin, “Extraordinary diseases require extraordinary solutions” (173).

### High Development Costs and Logistics of Mass-Production

It normally takes decades for a vaccine to be developed and licensed for use. However, the race is on to develop a SARS-CoV-2 vaccine for worldwide distribution in an unparalleled timeframe to vaccinate the world's population and provide widespread herd immunity. This is already being facilitated by rapid progression through the normally slow bureaucratic regulatory and approval processes and unprecedented worldwide collaboration between governments, universities, pharmaceutical companies and philanthropic organizations; such efforts must continue. In addition, ensuring good public communication regarding the safety and effectiveness of the vaccine will be key to gaining public trust and acceptance of a vaccine that could be seen as rushed. Additionally, having carefully designed post-marketing (phase 4) surveillance is vital to detect rare or unexpected safety signals and AESI which may only be detected once the vaccine is rolled out on a mass scale.

A key player in global access to a SARS-CoV-2 vaccine is the not-for-profit Coalition for Epidemic Preparedness Innovations (CEPI) which is working with global health authorities and vaccine developers worldwide to support SARS-CoV-2 vaccine development (9, 174). CEPI was founded in 2017 with the consensus that new and sustainable models of partnership are needed to respond to worldwide infectious diseases threats. Founding members include the Bill & Melinda Gates Foundation (BMGF), Wellcome trust UK, World Economic Forum, India Department of Biotechnology and the Government of Norway. CEPI's goal is not just to advance development, but to also advance licensing, manufacturing, delivery and stockpiling of vaccines once an effective vaccine has been developed (175). It has raised approximately $1.4 billion for SARS-CoV-2 vaccine development and initiated a series of partnerships in a bid to advance frontrunner candidate SARS-CoV-2 vaccines. CEPI is investing across a range of vaccine technologies including Novavax's nanoparticle vaccine (NVX-CoV2373 and Matrix-M™), Clover Biopharma's S-trimer protein-based vaccine (SCB-2019), University of Queensland's molecular clamp S protein vaccine, University of Oxford's adenovirus vector vaccine (ChAdOx1 nCoV-19), Inovio's DNA plasmid vaccine (INO-4800) and Moderna's and Curevac's mRNA vaccines (176). Many other government and philanthropic organizations are injecting large sums of money into the effort to develop effective SARS-CoV-2 vaccines. The European Commission has registered an impressive $8 billion in donations toward the development and deployment of vaccines, treatments and diagnostics against SARS-CoV-2 (177). The US government agency Biomedical Advanced Research and Development Authority (BARDA) has pledged almost $500 million to accelerate the development of Moderna's mRNA-1273 vaccine, with phase 2 trials expected to begin soon (178). BARDA has also funded Janssens's adenovirus 26 (Ad26) i.n. vaccine, Astra Zeneca's AZD1222 vaccine (formerly ChAdOx1 nCoV19), Merck and IAVI's rVSVG-CoV2 and Sanofi's recombinant SARS-CoV-2 protein vaccine and Novavax's nanoparticle Matrix M adjuvant vaccine (179), demonstrating the range of vaccine strategies being supported. Importantly, these phase 2/3 studies are being conducted in countries with high disease burden (e.g., North and South America and South Africa), so the vaccine efficacy (VE) and safety results can be obtained as rapidly as possible whilst meeting the vaccine regulators' requirements.

The logistics of scaling up to produce the billions of doses required to immunize the world's population is an onerous task. It is not yet clear how long immunity will last and therefore whether booster doses or annual immunization will be required. Furthermore, multiple initial doses may be needed and the vaccine may have to change as the virus evolves naturally. BARDA have pledged that they will scale up to 300 million annual SARS-CoV-2 vaccine doses in the US each year under Operation Warp Speed, while BMGF have recently awarded $5 million to INOVIO's DNA based vaccine INO-4800 with the intention of providing over 1 million doses by the end of 2020. Novavax has completed phase 1 studies and is progressing rapidly to phase 2 studies, with the aim of producing up to 100 million doses by the end of 2020 and entering large-scale manufacturing of billions of doses in 2021 (180). CureVac has a GMP-certified production facility that can produce 10 million doses of their mRNA vaccine in a single production run and 300 million doses of AZD1222 (ChAdOx1 nCoV-19) will be available by July 2021 (181).

### Target Populations and Worldwide Delivery

Even if sufficient SARS-CoV-2 vaccine doses can be manufactured, worldwide delivery presents another major logistic and financial hurdle. Storage requirements will be enormous and the vaccine may need to be either frozen or refrigerated, presenting cold-chain issues. The decision about whether to prepare the vaccine in single-dose or multi-dose vials will impact manufacturing, storage, delivery and potential infection risks (182). Infrastructure and manpower will be required to distribute and administer the vaccine. Equity will be a major issue since richer countries may procure the vaccine for their citizens while poorer countries may not be able to afford it. CEPI is negotiating global access upfront in order to ensure equitable access, hopefully averting “vaccine nationalism” (183). In early May, WHO launched the Access to COVID-19 Tools (ACT) Accelerator, which aims to handle the logistics of vaccine procurement and allocation (184). COVAX, co-led by CEPI, GAVI and WHO, is the vaccine pillar of ACT charged with accelerating vaccine development and manufacture and guaranteeing fair and equitable worldwide access. In an open letter, more than 140 world leaders have united and called for a SARS-CoV-2 vaccine to be freely available to all people in all countries of the world in what they call “The People's Vaccine,” which is surely the fairest way to tackle this unprecedented global pandemic (185). By contrast, pneumococcal conjugate vaccine which has been available for 20 years, is still not included in the immunization programs of many countries due to lack of affordability, many children are left unprotected and about half a million deaths from pneumococcal disease each year. Initially, as vaccine production commences, it will not be possible to deliver a SARS-CoV-2 vaccine to the entire world population and it is anticipated that key vulnerable populations will need to be targeted. These would likely include healthcare workers, the elderly and those with significant risk factors such as those with co-morbidities or the immunocompromised. It might even be used as a travel vaccine as borders re-open across the world, but is unlikely to be incorporated into infant schedules for some time given the very low risk of severe disease in that age group and time needed to conduct pediatric studies.

## Concluding Remarks

While the world eagerly awaits effective SARS-CoV-2 vaccines as the solution to ending this pandemic, it should be borne in mind that there are many caveats even if robust SARS-CoV-2-specific nAbs, CD4^+^ and CD8^+^ T cell responses can be induced by vaccination. The number of doses and dosing frequency will need to be determined, and, particularly if repeated or annual vaccination is required, the financial burden and logistics of delivery will have to be supported. A better understanding of what level of nAbs correlate with protection and development of standardized viral neutralization along with other assays to compare vaccine candidates is eagerly awaited. Potential disease enhancement and other theoretical safety concerns related to each type of vaccine need to be understood and carefully monitored for, while potential suboptimal immunity may need to be overcome. Certain vulnerable populations may respond poorly to vaccination, or the vaccine may predominantly protect against severe disease, rather than infection. If T cells prove critical for protection, it will be necessary to ensure that the included T cell epitopes are recognized by enough HLA types to ensure worldwide coverage. Scale-up of production to billions of doses and delivery to all regions of the world is a massive logistic challenge. In the short-term the strategy will likely need to be targeted vaccination toward those most at risk (e.g., healthcare workers) with the strategy reviewed as vaccine production increases. On the positive side, given the multiple candidates being developed, there is considerable optimism that some of the vaccines currently in trials will prove effective and be available for use in 2021 with delivery scaled-up thereafter.

## Author Contributions

KF proposed the project and coordinated the study group. KF, NC, and FR wrote the first draft. EB, MG, AK, KM, BT, and SW reviewed and edited the manuscript, provided comments, and suggested references substantially contributing to the content. All authors approved the final submitted version of the manuscript.

## Conflict of Interest

KF has served on advisory boards for Sanofi Pasteur and Seqirus in the last 5 years. BT is on the advisory board for CSL Behring and has received research funding from Sanofi Pasteur, MSD, and speaker fees from Gilead and Janssen. SW is an investigator on an MSD vaccine trial but receives no direct funds from MSD. The remaining authors declare that the research was conducted in the absence of any commercial or financial relationships that could be construed as a potential conflict of interest.

## Abbreviations

Abs: antibodies
ACE2: angiotensin-converting enzyme 2
ACT: Access to COVID-19 Tools
ADCA: antibody-dependent complement activation
ADCC: antibody-dependent cellular cytotoxicity
ADCP: antibody-dependent cellular phagocytosis
ADE: antibody dependent enhancement
AESI: adverse events of special interest
APC: antigen presenting cell
ARDS: acute respiratory distress syndrome
BARDA: Biomedical Advanced Research and Development Authority
BCG: bacillus Calmette-Guérin
BMGF: Bill & Melinda Gates Foundation
CEPI: Coalition for Epidemic Preparedness Innovations
COVID-19: coronavirus disease of 2019
CP: convalescent plasma
CTL: cytotoxic T lymphocyte
DAMPs: damage-associated molecular patterns
DC: dendritic cell
E protein: envelope protein
ELISpot: enzyme linked immunospot
GSCF: granulocyte colony-stimulating factor
HLA: human leukocyte antigen
HR1/2: heptad repeat fusion regions 1/2
IFN: interferon
IL: interleukin
IP-10: interferon-inducible protein 10
M protein: membrane protein
MCP-1: Monocyte chemoattractant protein-1
MDA5: melanoma differentiation-associated protein 5
MERS: Middle East respiratory syndrome
MIP-1α: macrophage inflammatory protein-1α
MPL: monophosphoryl lipid A
MVA: modified vaccinia Ankara
N protein: nucleocapsid protein
nAbs: neutralizing antibodies
NK cells: natural killer cells
OAS: original antigenic sin
PAMPs: pattern associated molecular patterns
PRRs: pattern recognition receptors
RBD: receptor binding domain
RIG-I: retinoic acid-inducible gene 1
RSV: respiratory syncytial virus
SARS: severe acute respiratory syndrome
SARS-CoV-1: severe acute respiratory syndrome coronavirus-1
SARS-CoV-2: severe acute respiratory syndrome coronavirus-2
S glycoprotein: spike glycoprotein
T_FH_: follicular helper T cell
Th1/2/17: T helper 1/2/17
TLR: Toll-like receptor
TNF: tumor necrosis factor
Tregs: regulatory T cells
VAERD: vaccine associated enhanced respiratory disease
VE: vaccine efficacy
VLP: virus like particle
VSV: vesicular stomatitis virus.
